# Comparing empirical kinship derived heritability for imaging genetics traits in the UK biobank and human connectome project

**DOI:** 10.1016/j.neuroimage.2021.118700

**Published:** 2021-11-02

**Authors:** Si Gao, Brian Donohue, Kathryn S. Hatch, Shuo Chen, Tianzhou Ma, Yizhou Ma, Mark D. Kvarta, Heather Bruce, Bhim M. Adhikari, Neda Jahanshad, Paul M. Thompson, John Blangero, L. Elliot Hong, Sarah E. Medland, Habib Ganjgahi, Thomas E. Nichols, Peter Kochunov

**Affiliations:** a Department of Psychiatry, Maryland Psychiatric Research Center, School of Medicine, University of Maryland, Baltimore, MD, United States; b Department of Epidemiology and Biostatistics, University of Maryland, College Park, MD, United States; c Department of Neurology, Imaging Genetics Center, Mark & Mary Stevens Institute for Neuroimaging and Informatics, Keck School of Medicine, University of Southern California, Marina del Rey, CA, United States; d University of Texas Rio Grande Valley, Harlingen, TX, United States; e QIMR Berghofer Medical Research Institute, Queensland, Australia; f Department of Statistics, Big Data Science Institute, University of Oxford, Oxford, United Kingdom

**Keywords:** Heritability, Imaging genetics, Computational methods, Pedigree, FPHI, GCTA

## Abstract

Imaging genetics analyses use neuroimaging traits as intermediate phenotypes to infer the degree of genetic contribution to brain structure and function in health and/or illness. Coefficients of relatedness (CR) summarize the degree of genetic similarity among subjects and are used to estimate the heritability – the proportion of phenotypic variance explained by genetic factors. The CR can be inferred directly from genome-wide genotype data to explain the degree of shared variation in common genetic polymorphisms (SNP-heritability) among related or unrelated subjects. We developed a central processing and graphics processing unit (CPU and GPU) accelerated Fast and Powerful Heritability Inference (FPHI) approach that linearizes likelihood calculations to overcome the ~*N*^2–3^ computational effort dependency on sample size of classical likelihood approaches. We calculated for 60 regional and 1.3 × 10^5^ voxel-wise traits in *N* = 1,206 twin and sibling participants from the Human Connectome Project (HCP) (550 M/656 F, age = 28.8 ± 3.7 years) and *N* = 37,432 (17,531 M/19,901 F; age = 63.7 ± 7.5 years) participants from the UK Biobank (UKBB). The FPHI estimates were in excellent agreement with heritability values calculated using Genome-wide Complex Trait Analysis software (*r* = 0.96 and 0.98 in HCP and UKBB sample) while significantly reducing computational (10^2–4^ times). The regional and voxel-wise traits heritability estimates for the HCP and UKBB were likewise in excellent agreement (*r* = 0.63–0.76, *p* < 10^−10^). In summary, the hardware-accelerated FPHI made it practical to calculate heritability values for voxel-wise neuroimaging traits, even in very large samples such as the UKBB. The patterns of additive genetic variance in neuroimaging traits measured in a large sample of related and unrelated individuals showed excellent agreement regardless of the estimation method. The code and instruction to execute these analyses are available at www.solar-eclipse-genetics.org.

## 1. Introduction

Big data research initiatives - including the Human Connectome Project (HCP) and UK Biobank (UKBB) - collect comprehensive multimodal neuroimaging datasets and allow researchers to quantify genetic and environmental risk and protective factors that affect human brain in health and illness (Glasser et al., 2013; Van Essen et al., 2013). Genetic variance accounts for a significant proportion (20–90%) of functional and structural variability in human brain (Adhikari et al., 2017; Hulshoff Pol et al., 2006; Pennington et al., 2000; Pfefferbaum et al., 2000; Thompson et al., 2010). Heritability (*h*^*2*^) is defined as the degree of phenotypic variance explained by the additive genetic variance among participants. Classically, heritability is calculated using variance component models that use coefficients of relatedness (CR) to represent the shortest self-reported ancestral path for a pair of individuals as the degree of genetic variance shared among individuals. CR can also be calculated empirically from high-throughput genome-wide single nucleotide polymorphism (SNP) data, in which case the heritability measures the proportion of the observed variation explained by common SNPs (SNP-*h*^*2*^) (Kochunov et al., 2019a; Ramstetter et al., 2017; Speed et al., 2017; Toro et al., 2014; Wood et al., 2014; Yang et al., 2010). In family samples, the empirical CR tracks closely with self-reported values but provides more accurate estimates of heritability (Kochunov et al., 2019a). The SNP-*h*^*2*^ can also be calculated in samples of unrelated individuals based on the phenotypic variance explained by small amounts of genetic similarity shared among participants (Yang et al., 2010). Here, we performed two sets of analyses: We first evaluated a novel Fast and Powerful Heritability Inference (FPHI) approach that accelerates classical variance component models using algorithmic and hardware approaches and compared the measurements to that of a commonly used SNP-*h*^*2*^ approach implemented in the Genome-wide Complex Trait Analysis (GCTA) software (https://cnsgenomics.com/software/gcta/). We compared heritability estimates for complex polygenic neuroimaging traits in a twin-and-siblings sample collected by HCP and mainly unrelated sample provided by UKBB. We finally showed a good agreement in heritability estimates measured in UKBB and these reported by large meta-and-mega analyses performed by Enhancing Neuro Imaging Genetics through Meta-Analysis (ENIGMA) studies (Jahanshad et al., 2013; Kochunov et al., 2014).

We first set out to study an agreement in heritability estimates using empirical CR values by our novel FPHI approach implemented within the SOLAR-Eclipse software (www.solar-eclipse-genetics.org) and SNP-heritability measured using the GCTA software which pioneered the SNP-*h*^*2*^ measurements. SNP-*h*^*2*^ refers to the proportion of phenotypic variance explained by the individual variances in the SNP data collected from genotyping arrays. SNP-*h*^*2*^ values can be calculated using classical variance component such as these implemented in FPHI or fitting the linear model across all SNP as implemented in GCTA. It can also be calculated using linkage disequilibrium (LD) score regression (LDSR) approaches that use summary statistics for trait from a genome-wide association study analysis (GWAS) (Speed et al., 2012, 2017). All approaches have advantages and shortcomings regarding estimation bias, robustness, and computational efficiency. In this study, we did not consider LDSR because these analyses require performing GWAS analysis for a trait. LDSR analyses are practical when the summary statistics are already available. However, performing GWAS while correcting for the relatedness within a sample is a computationally formidable task, especially for voxel-wise traits. Both FPHI and GCTA uses algorithmic accelerations to make SNP-*h*^*2*^ calculation practical in the absence of GWAS summary statistics.

Classical heritability analyses use variance models that partition the phenotypic variance into the additive genetic and environmental components (See supplement for details) (Nayor et al., 2021). These models rely on the *N* × *N* matrix of CR values (where *N* is the sample size), known as the pedigree or kinship matrix to map the sharing of genetic variance among subjects. Traditionally, CR values were fixed to the theoretical values of the expected degree of autosomal genomic sharing for a given kinship type: 1 for the similarity with oneself, or with a monozygotic twin; $\frac{1}{2}$ for parents, full siblings and dizygotic twins; $\frac{1}{4}$ for grandparents or half-siblings; 1/8 for cousins; and 0 for unrelated individuals. However, with the development of genome-wide genotyping technologies, CR values can also be measured empirically by quantifying the similarity across genome or chromosomal SNP sets among the study participants. Comparisons of traditional versus empirical CR values show that there is variation in shared genetic variance around the traditional estimates and that seemingly unrelated individuals can have a non-zero degree of shared genetic variance (Kochunov et al., 2019a; Visscher et al., 2006, 2007). Neuroimaging traits have a complex polygenic architecture, and more precise estimation of the CR values can improve statistical power for genetic analyses (Kochunov et al., 2019a).

The general formulation of the classical variance component model, such as implemented in SOLAR-Eclipse/FPHI software, allows for the use of empirical CR matrix estimates of the genetic relatedness across a wide-range of related individuals (Kochunov et al., 2019a; Zaitlen et al., 2013). Here, we evaluated the agreement among SNP-*h*^*2*^ values calculated by FPHI and by the GCTA software that was specifically developed for SNP-based heritability (Visscher et al., 2006, 2007). Our goal was to show that heritability values derived by FPHI and GCTA closely agree using data from samples such as the HCP and UKBB. However, the GCTA approach may not scale readily to large samples such as the UKBB due to its computational complexity and non-linear dependance of computational time versus pedigree size. The SOLAR-Eclipse FPHI approach uses software and hardware optimizations, including parallel CPU/GPU computing, to linearize likelihood estimation and achieves ~10^5–6^ performance improvement versus classical iterative likelihood approaches (Nayor et al., 2021). Here, we show that FPHI approach makes practical calculation of SNP-*h*^*2*^ values for calculation of high-resolution voxel-wise heritability maps.

**Fig. 1.**
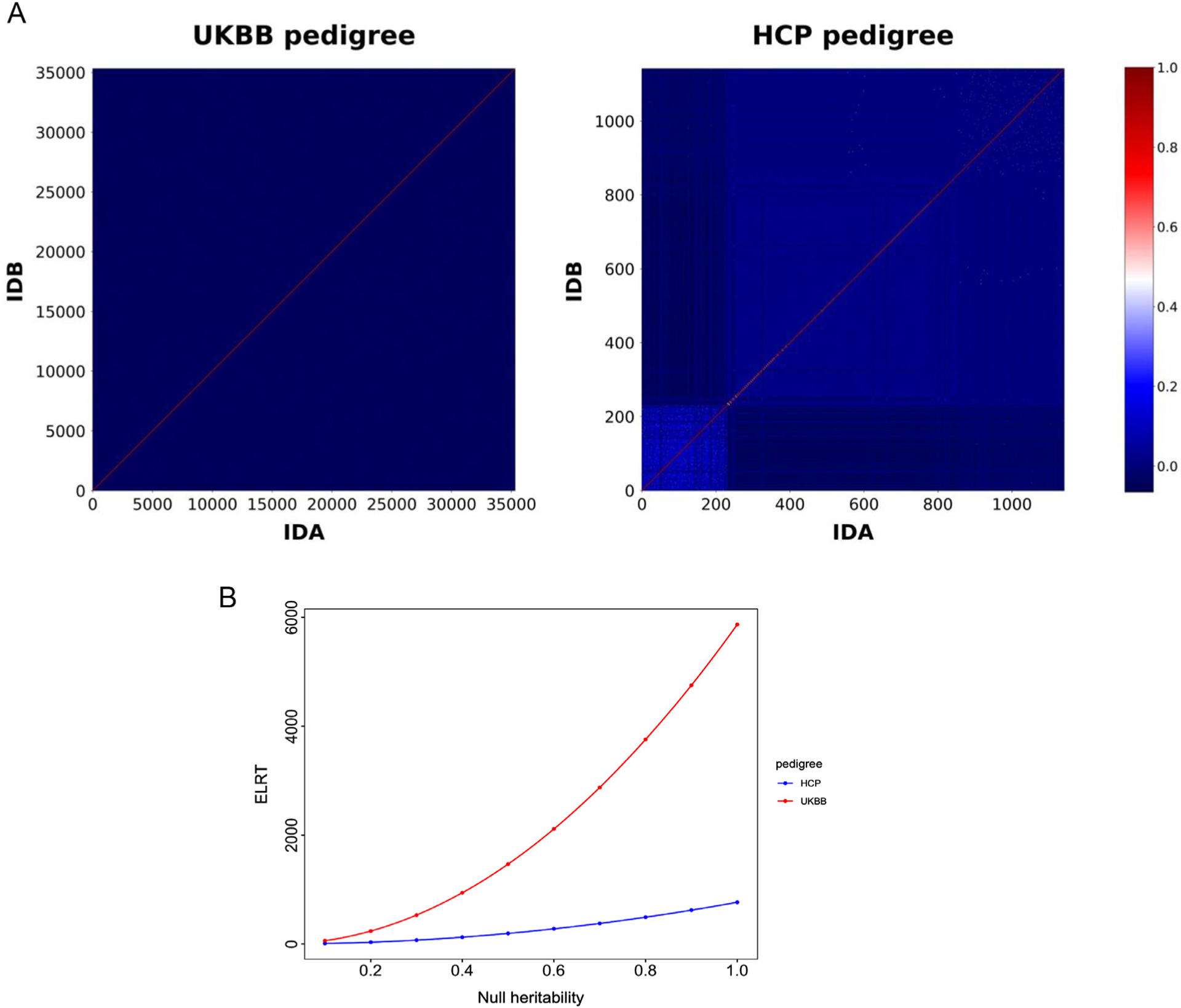
**A.** Heatmaps of the UKBB and HCP pedigrees. The heatmaps present CR values between individuals in pedigrees. The color bar reflects negative and positive CR values in the heatmaps. The diagonal is CR between the same individual. **B.** The ELRT power curves for the HCP and UKBB samples. The blue and red dots indicate expected likelihood ratio test (ELRT) at specific null-heritability values for the UKBB and HCP, respectively.

SOLAR-Eclipse uses a Weighted Allelic Correlation (WAC) approach to calculate the empirical CR. The WAC–CR values provided more stable empirical heritability measures than those from other methods, including self-reported CR, although the differences were minor (Kochunov et al., 2019a). The WAC was developed to study the “missing heritability” of complex phenotypes and produces CR values that are weighted by minor allele frequency (MAF) using a parameter, *α*, with assigned values of 1, −1, or 0 (Speed et al., 2012, 2017). A weighting of *α* = 1 calculates CR by up-weighting on common variants, whereas a weighting of *α* = −1 up-weights CR on the low MAF variants. The weighting of *α* = −1 was recommended for human studies based on empirical findings and simulations that show that it up-weights CR on the low MAF variants, reduces the bias and increases the precision of heritability estimation, while other *α* were found more appropriate for animal or plants genetics studies (Speed et al., 2012, 2017). However, in our prior research, we found very minor differences in the heritability estimates obtained with different *α* settings in imaging genetics analyses (Kochunov et al., 2015). The WAC approach produces a very dense *N* × *N* (where *N* is the sample size) pedigree matrix (Fig. 1). This is a computational challenge for traditional maximum likelihood estimate (MLE) calculation approaches. The MLE procedure requires multiple inversions of this matrix leading to an *N*^2–3^ computational complexity problem which makes Big Data analyses a formidable challenge (Blangero et al., 2013).

In this study we present novel algorithmic developments that address a major roadblock to enable imaging genetics analyses in datasets as large as *N* > 35,000 based on our previous work on linearizing likelihood calculation (Blangero et al., 2013). We demonstrate that the classical quantitative genetics analyses can now be practical in large and inclusive datasets of unrelated individuals. We describe algorithmic solutions to take advantage of Central and Graphics Processing Units (CPU and GPU) computing. Our proposed method leads to improvements in the computational times while maintaining excellent agreement with results from other software (Blangero et al., 2013; Kochunov et al., 2019a, 2019b). Here, we demonstrated that empirical heritability measurement can be achieved in seconds using modern computational hardware, even in samples as large as the UKBB.

## 2. Materials and methods

### 2.1. Participants

#### Human Connectome Project.

Heritability and genetic correlation analyses were performed on brain MRI scan data from *N* = 1206 (550 M/656 F; age = 28.8 ± 3.7 years) healthy individuals in the Human Connectome Project (HCP) (humanconnectome.org) for whom imaging and genetic data were released after passing the HCP quality control and assurance standards (Marcus et al., 2013). Details of this release may be found at (https://www.humanconnectome.org/study/hcp-young-adult/document/1200-subjects-data-release). Participants in the HCP study were recruited from the Missouri Family and Twin Registry of individuals born in Missouri (Van Essen et al., 2013). The full set of inclusion and exclusion criteria are detailed elsewhere (Van Essen et al., 2013). All participants provided written informed consent on forms approved by the Institutional Review Board of Washington University in St. Louis.

#### UK BioBank.

The UK BioBank (UKBB) dataset included *N* = 37,432 individuals (17,531 M/19,901 F; age = 63.7 ± 7.5 years) whose imaging and genetic data were released from 2015 to 2021. The full set of inclusion and exclusion criteria are detailed elsewhere (Manolio et al., 2012). All participants provided written informed consent.

### 2.2. Genotyping

We used genotyping data provided by HCP and UKBB projects with minimal post-processing as recommended by GCTA software manual. The genotyping data for the HCP is available through the dbGAP database (https://www.ncbi.nlm.nih.gov/projects/gap/cgi-bin/study.cgi?study_id=phs001364.v1.p1). Briefly, all participants were genotyped using the Illumina Multi-Ethnic Global Array (MEGA) SNP-array. This array provides extended coverage for European, East Asian, and South Asian populations. Overall, 1,580,642 SNPs satisfied the following quality control exclusion criteria: MAF < 1%, genotype call rate < 95%, and Hardy–Weinberg equilibrium < 1 × 10^−6^.

Genotyping data for the UKBB was downloaded as version 3 imputed data from the UKBB showcase website. The protocol for genotyping, imputation and quality control is described in sections of the UK Biobank documentation (https://biobank.ndph.ox.ac.uk/showcase/showcase/docs/genotyping_qc.pdf) and (https://biobank.ndph.ox.ac.uk/showcase/showcase/docs/impute_ukb_v1.pdf). In summary, all participants were genotyped using the UKBB Axiom array from Affymetrix and imputed using Haplotype Reference Consortium (HRC) and UK10K haplotype resource. Overall, there were 8,521,984 SNPs remaining after the same exclusion criteria as used for HCP data.

### 2.3. Neuroimaging traits

We selected traits from four neuroimaging domains: cortical gray matter thickness, subcortical gray matter volumes, fractional anisotropy FA values of water diffusion measured for regions of interest (Table S1), and voxel-wise FA values for the whole-brain skeleton.

#### HCP imaging data collection and preprocessing.

The HCP data was collected at Washington University, St. Louis, using a customized Siemens Magnetom Connectome 3 Tesla scanner with a 100 mT/m maximum gradient strength and a 32-channel head coil. Details on the scanner, image acquisition, and reconstruction are provided elsewhere (Ugurbil et al., 2013) and found online at (https://www.humanconnectome.org/study/hcp-young-adult/document/1200-subjects-data-release). Diffusion data was collected using a single-shot, single refocusing spin-echo, echo-planar imaging sequence with 1.25 mm isotropic spatial resolution (TE/TR = 89.5/5520 ms, FOV = 210 × 180 mm). Three gradient tables of 90 diffusion-weighted directions and 6 *b* = 0 images each, were collected with right-to-left and left-to-right phase encoding polarities for each of the three diffusion weightings (*b* = 1000, 2000, and 3000 s/mm^2^). The diffusion data were then processed using the Enhancing Neuro Imaging Genetics through Meta-Analysis (ENIGMA) pipeline for structural and diffusion tensor imaging, including skeletonized voxel-wise FA values (Jahanshad et al., 2013).

#### UKBB imaging data collection and preprocessing.

The UKBB imaging data were collected using three sites each equipped with a Siemens Skyra 3T scanner and 32-channel RF head coil with high resolution T1-weighted (resolution = 1 × 1 × 1 mm, FOV = 208 × 256 × 256, duration = 5 min, 3D MPRAGE, sagittal, in-plane acceleration iPAT = 2, prescan-normalize). Diffusion data was acquired with the following parameters: a resolution = 2 × 2 × 2 mm and two diffusion-weighted shells with all 100 distinct diffusion-encoding directions, 5 *b* = 0 images, 50x *b* = 1000 and 2000 s/mm^2^, FOV = 104 × 104 × 72, and a 7-minute duration. The data were extracted using the UKBB workflow and processed using the UKBB processing pipeline (https://git.fmrib.ox.ac.uk/falmagro/UK_biobank_pipeline_v_1).

We used average regional and skeletonized imaging data provided by the UKBB. The skeletonized data were extracted using the UKBB workflow. More information on the scanner, image acquisition, and processing are all recorded in the UKBB Brain Imaging Documentation (https://biobank.ctsu.ox.ac.uk/crystal/crystal/docs/brain_mri.pdf) (Alfaro-Almagro et al., 2018; Miller et al., 2016). All data were preprocessed prior to FPHI and GCTA analyses to reduce potential confounding of different approaches these tools may use for regression the effects of covariates. We used SOLAR-Eclipse mega-analysis data normalization pipeline to regress effects of age, sex and scan site (for UKBB data) and saving the residuals (Kochunov et al., 2014). This was followed with the inverse normal transformation was used to ensure the multivariate normal distribution of the traits (Kochunov et al., 2014, 2019a).

### 2.4. Assessment of empirical relatedness

SOLAR-Eclipse uses CR (r_i,j_) (twice the coefficients of kinship) to represent the probability that two alleles from individuals *i* and *j* are identical by descent. The coefficient of relationship is a function of identity by descent sharing statistics, r_i,j_ = *π*_1i,j_/2 + *π*_2i,j_, where *π*_1i,j_ and *π*_2i,j_ are the probabilities that two individuals share one and two alleles through a common ancestry. Empirical r_i,j_ were calculated using methods implemented in the SOLAR-Eclipse software (www.solar-eclipse-genetics.org). The *pedifromsnps* function uses the allelic data stored in a PLINK file as the input and produces a pedigree file. We calculated empirical r_i,j_ using weighted allelic correlation (WAC) (Hayes et al., 2009). This function is implemented for GPU computing in the *gpu_pedifromsnps* function. Relatedness was calculated using Eq. (1):

(1) $$\phi_{ij}=\frac{1}{m}\overset{m}{\underset{k=0}{\sum }}\frac{\left(SN\;P_{ik}-2\mu_{k}\right)\left(SN\;P_{jk}-2\mu_{k}\right)}{2\mu_{k}\left(1-\mu_{k}\right)}$$

where *ϕ*_*ij*_ is the genetic relationship matrix (GRM)/empirical kinship matrix value between individual *i* and individual *j*. m is the total number of SNP loci that are not missing values for both individual *i* and individual *j*. *SNP*_*ik*_ and *SNP*_*jk*_ are allelic scores (0, 1 or 2) for the *k*-th SNP in individuals *i* and *j*. *μ*_*k*_ is the frequency of the *k*-th major allele.

### 2.5. Comparison of pedigree power: expected likelihood ratio test (ELRT)

The ELRT method is used by SOLAR-Eclipse software to evaluate the statistical power of a pedigree for heritability analysis and to compare power between two pedigrees. This function is based on the functionality proposed by (Blangero et al., 2013) and further generalized by (Raffa and Thompson, 2016). The ELRT is defined as the expectation of twice the difference of the log-likelihoods evaluated at the true parameter and several different null-parameter values, respectively (Raffa and Thompson, 2016). It uses Taylor series approximations to summarize the relatedness in a pedigree to accurately approximate the expectation of the likelihood ratio test and expected confidence interval widths (Raffa and Thompson, 2016).

### 2.6. Analysis of additive genetic variance: heritability

The algorithms used to estimate variance components employ a variance decomposition approach based on an extension of the strategy developed by (Amos, 1994) and optimized for parallel computing and coded as the *fphi* function. The multivariate normal covariance matrix Ω for a pedigree of individuals is given by Eq. (2):

(2) $$\Omega =2\sigma_{g}^{2}\Phi +\sigma_{e}^{2}I$$

where Φ is the empirical kinship matrix among all participants, *σ*_*e*_^*2*^ is the variance caused by environmental effects and measurement errors, and *I* is an identity matrix under the assumption that all environmental effects are uncorrelated among family members.

Heritability (*h*^*2*^) is the proportion of the total phenotypic variance (*σ*_*p*_^*2*^) that can be explained by the additive effects of genes (*σ*_*g*_^*2*^):

(3) $$h^{2}=\frac{\sigma_{g}^{2}}{\sigma_{P}^{2}}$$

The *fphi* function uses algorithmic developments to reduce the computational burden of heritability measurements (see supplement). This approach uses eigenvalue decomposition of the empirical kinship matrix, Φ (Blangero et al., 2013), and then performs one-step asymptotically unbiased MLE estimation (Ganjgahi et al., 2015). The variance parameters are estimated by comparing the observed phenotypic covariance matrix with the covariance matrix predicted by kinship (Almasy and Blangero, 1998). Significance of heritability is assessed using a likelihood-ratio test, which compares the maximum likelihood with the likelihood estimation in which *σ*_*g*_^*2*^ is constrained to zero in the model. Twice the difference between the log-likelihoods of these models yields a test statistic, which is a 1/2:1/2 mixture of an asymptotic *χ*^*2*^ distribution with 1°-of-freedom and a point mass at zero.

### 2.7. GCTA analysis

We compared the heritability values estimated using FPHI to those estimates using the restricted MLE approach used within GCTA (Lee et al., 2011; Yang et al., 2010). The GCTA approach estimates the proportion of the variance of the phenotype that is explained by the genome-wide genotypic data, or in this case, SNPs. Specifically, the variance is estimated by fitting the following linear mixed model, in Eq. (4):

(4) y=Xβ+Φu+ε

(5) $$\upsilon ar(y)=\sigma_{g}^{2}G+\sigma_{\varepsilon }^{2}I$$

where *y* is the vector of phenotypes, *β* is the vector of fixed effects of covariates to be adjusted, Φ is the matrix of the coefficients of relatedness and *u* is the vector of random effects from SNPs with $u\sim n\left(0,\;\sigma_{u}^{2}I\right)$, *ε* is the vector of residual effects with $\varepsilon \sim n\left(0,\;\sigma_{\varepsilon }^{2}I\right)$, ***G*** = *ϕϕ*′/m, where m is the number of SNPs.

GCTA also estimates the GRM using the WAC approach (Eq. (1)). The variance explained by the genotypic data used in the analyses, $\sigma_{g}^{2}=m\sigma_{u}^{2}$, is estimated using the genomic-relatedness-based restricted maximum likelihood (GREML) approach. The heritability can then be estimated as: $h^{2}=\sigma_{g}^{2}/\left(\sigma_{g}^{2}+\sigma_{\varepsilon }^{2}\right)$, the proportion of total phenotypic variance that is due to additive genetic effects. The iterative REML approach performs an inversion of the Φ matrix at every iteration. Φ is a dense matrix and the computational complexity of this operation is a function of ~*N*^2–3^, where *N* is the number of subjects. This computational effort of iterative likelihood calculations becomes non-trivial for very large-scale studies such as the UKBB (*N* = 500,000 and growing).

### 2.8. Timing analysis: FPHI versus GCTA and FPHI CPU versus FPHI GPU

Large-scale imaging genetic analyses such as voxel-wise heritability calculations in large datasets, such as the UKBB, may benefit from modern computational hardware. The highly parallel and non-iterative nature of the SOLAR-Eclipse FPHI algorithms calls for efficient implementation using modern hardware optimized for massively parallel computations (see supplement). Here, we tested the timing of trait-wise analyses for FPHI and GCTA, and the voxel-wise analysis between CPU and GPU versions of the FPHI. The voxel-wise analyses were not tested with GCTA due to very long (estimated several years) calculation times. We used a Lenovo computer with 256 GB of RAM and equipped with a dual Intel Xeon Gold 6150 processor with 18 cores running at 2.7 GHz (36 cores in total) and a Tesla P100 GPU card with 3584 cores and 16 GB of GPU RAM.

## 3. Results

### 3.1. Empirical pedigrees: HCP and UKBB

As expected, the HCP pedigree had a higher average CR than that of the UKBB (Fig. 1A). However, ELRT analysis indicated that the UKBB pedigree had higher statistical power for heritability studies. The power of a pedigree is proportional to both the average relatedness among the subjects and the *N* and therefore the large UKBB sample provided more power than the HCP sample (Fig. 1B).

### 3.2. SOLAR-Eclipse vs GCTA

**Fig. 2.**
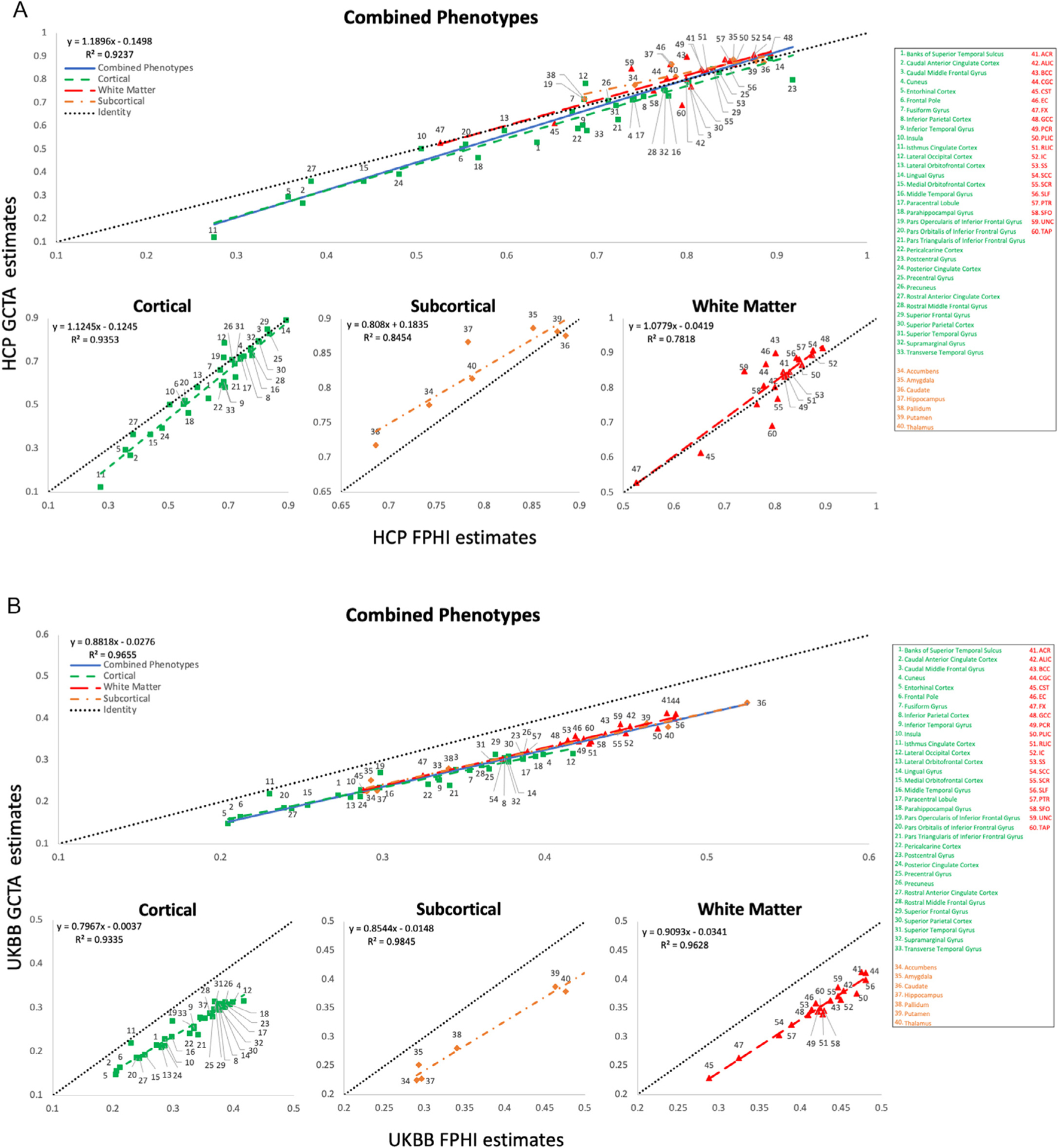

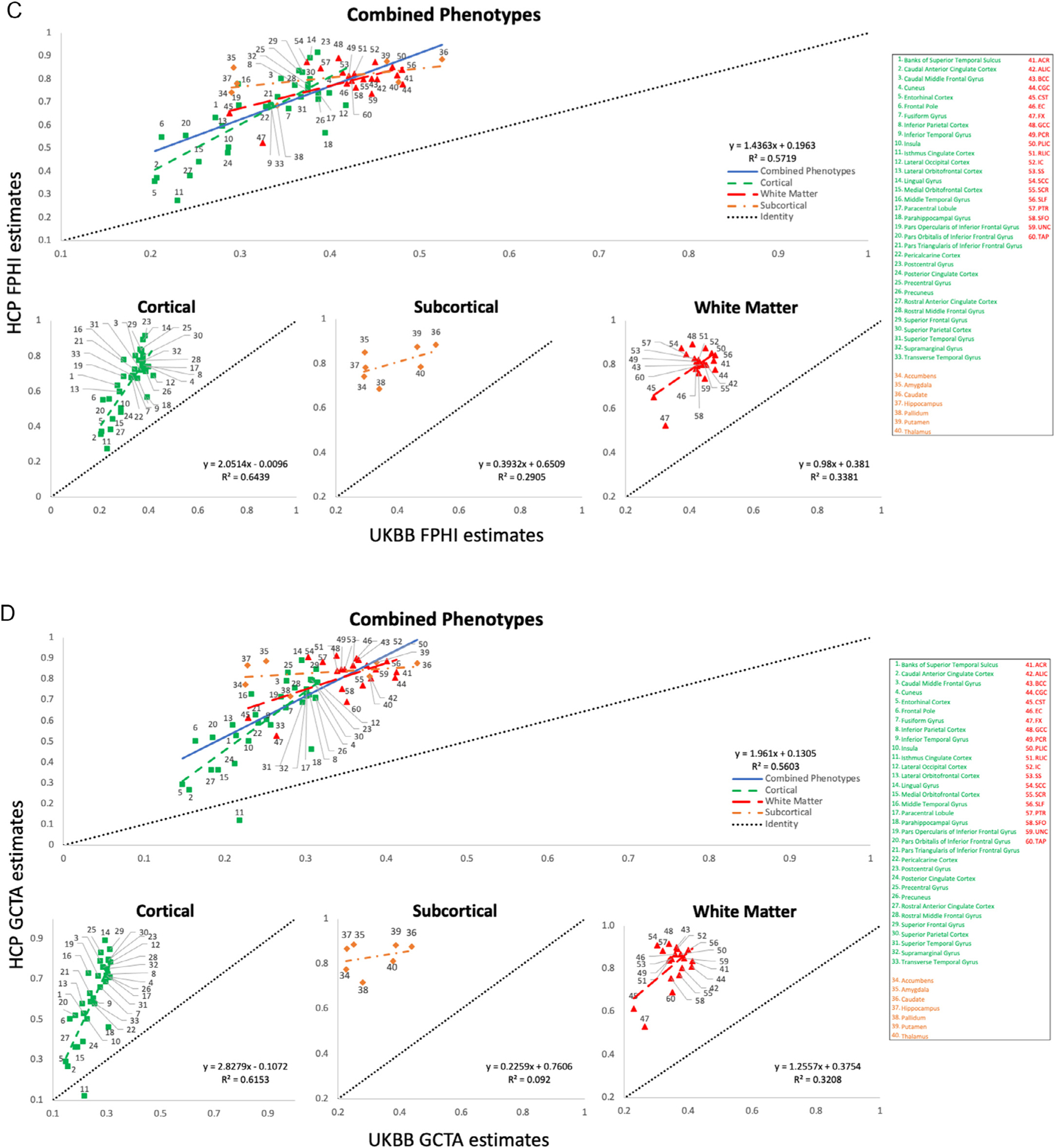
**A.** Scatter plot of the HCP FPHI estimates calculated using empirical kinship versus HCP GCTA estimates calculated using GREML for 60 neuroimaging phenotypes. Linear regression models were fitted to the HCP heritability estimates using the FPHI and GCTA methods, including fit lines, equations, and coefficient of determinations (R^2^). The blue solid line is an overall linear regression fit between two heritability methods across all phenotypes in the HCP. The green dashed lines, red dashed lines and orange dashed lines represent linear regression fits between two heritability methods in cortical thickness, white matter FA and subcortical volume, respectively. The black dashed lines are identity lines. **B.** Scatter plot of the UKBB FPHI estimates calculated using empirical kinship versus UKBB GCTA estimates calculated using GREML for 60 neuroimaging phenotypes. Linear regression models were fitted to the UKBB heritability estimates using the FPHI and GCTA methods, including fit lines, equations, and coefficient of determinations (R^2^). The blue solid line is an overall linear regression fit between two heritability methods across all phenotypes in the UKBB. The green dashed lines, red dashed lines and orange dashed lines represent linear regression fits between two heritability methods in cortical thickness, white matter FA and subcortical volume, respectively. The black dashed lines are identity lines. **C.** Scatter plot of the UKBB FPHI estimates calculated using empirical kinship versus the HCP FPHI estimates calculated using empirical kinship for 60 neuroimaging phenotypes. Linear regression models were fitted to the UKBB and HCP heritability estimates using the FPHI method, including fit lines, equations, and coefficient of determinations (R^2^). The blue solid line is an overall linear regression fit between two groups across all phenotypes. The green dashed lines, red dashed lines and orange dashed lines represent linear regression fits between two groups in cortical thickness, white matter FA and subcortical volume, respectively. The black dashed lines are identity lines. **D.** Scatter plot of the UKBB GCTA estimates calculated using GREML versus the HCP GCTA estimates calculated using GREML for 60 neuroimaging phenotypes. Linear regression models were fitted to the UKBB and HCP heritability estimates using the GCTA method, including fit lines, equations, and coefficient of determinations (R^2^). The blue solid line is overall linear regression between two groups across all tracts. The blue line is an overall linear fits regression between two groups across all phenotypes. The green dashed lines, red dashed lines and orange dashed lines represent linear regression fits between two groups in cortical thickness, white matter FA and subcortical volume, respectively. The black dashed lines are identity lines.

The scatter plots of the heritability estimates showed an excellent agreement (overall regression *r* = 0.96 and 0.98, *p* < 10^−10^) between the *h*^*2*^ values estimated from FPHI and GCTA in both the HCP and UKBB samples (Fig. 2A and B, Table S1; see supplement). The heritability estimates by FPHI and GCTA showed no significant differences in the HCP (average *h*^*2*^ = 0.72 ± 0.15 versus. 0.70 ± 0.18, paired *t*-test *p* = 0.1). However, the average FPHI *h*^*2*^ estimates were higher than GCTA-derived *h*^*2*^ values in the UKBB (average *h*^*2*^ = 0.36 ± 0.08 versus 0.29 ± 0.07, paired *t*-test *p* < 10^−10^).

### 3.3. Regional and voxel-wise heritability in the HCP versus UKBB

The regional heritability analyses showed good agreement between HCP and UKBB (Fig. 2C and D) when calculated using FPHI (overall linear regression *r* = 0.76, *p* < 10^−10^) and GCTA (overall linear regression *r* = 0.75, *p* < 10^−10^). However, the heritability estimates in UKBB were approximately 50% lower than those for HCP (average *h*^*2*^ = 0.36 ± 0.08 versus 0.72 ± 0.15, paired *t*-test *p* < 10^−10^).

The plot of voxel-wise heritability values of skeletonized FA values for 32,215 voxels that overlapped between UKBB and HCP skeletons is shown in Fig. S1 (see supplement). Overall, the regional pattern of heritability showed a good agreement (overall linear regression *r* = 0.76, *p* < 10^−10^). However, the voxel-wise heritability estimates in the UKBB sample were lower than those for HCP (average *h*^*2*^ = 0.16 ± 0.08 versus *h*^*2*^ = 0.25 ± 0.16, paired *t*-test *p* < 10^−10^ for UKBB and HCP, respectively).

### 3.4. Regional white matter heritability: UKBB versus. ENIGMA

**Fig. 3.**
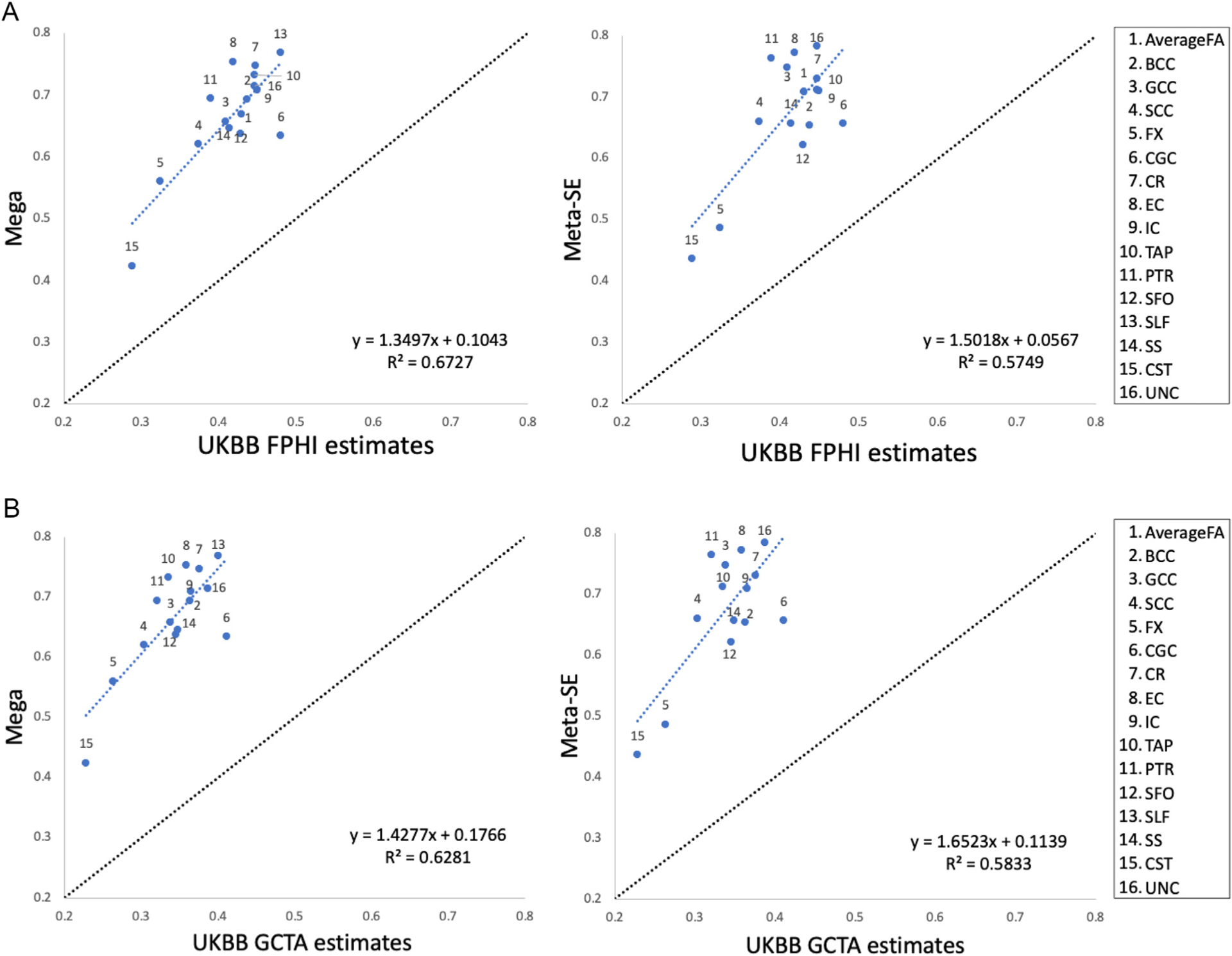
**A.** Scatter plot of the UKBB FPHI estimates versus ENIGMA for 16 white matter FA. Linear regression models were fitted to the heritability estimates from the FPHI and published heritability estimates from ENIGMA for 16 white matter phenotypes in the UKBB. The linear regression fits include fit lines, equations, and coefficient of determinations (R^2^). The black dashed lines are identity lines. **B.** Scatter plot of the UKBB GCTA estimates versus ENIGMA for 16 white matter FA. Linear regression models were fitted to the heritability estimates from the GCTA and published heritability estimates from ENIGMA for 16 white matter phenotypes in the UKBB. The linear regression fits include fit lines, equations, and coefficient of determinations (R^2^). The black dashed lines are identity lines.

ENIGMA has published regional white matter heritability meta- and mega- analytical multi-site estimates from a multi-site heritability analysis. The FPHI and GCTA heritability estimates for white matter tracts in UKBB showed good agreement with the published values (linear regression *r* = 0.76–0.82, *p* < 0.01) (Fig. 3A and B). The heritability estimates in the UKBB were approximately 60% of the *h*^*2*^ values reported in ENIGMA (average *h*^*2*^ = 0.42 ± 0.05 versus *h*^*2*^ = 0.67 ± 0.09, paired *t*-test *p* < 10^−10^ for UKBB and ENIGMA, respectively).

### 3.5. Timing of heritability analyses

FPHI-CPU analyses in the HCP required ~0.02 ± 0.01 s per trait versus 3.0 ± 0.10 s per trait for GCTA. The heritability analyses of regional phenotypes in the UKBB took about 1.1 ± 0.10 s per trait using FPHI-CPU and 2046 ± 470 s for GCTA.

The timing of voxel-wise analyses was limited to FPHI due to the long execution time of GCTA (estimated ~7 years for UKBB analyses). We performed a timing analysis for the CPU and GPU versions of FPHI in SOLAR-Eclipse. The FPHI-CPU voxel-wise heritability analyses took approximately 2 min for HCP and 22 h for UKBB. The FPHI-GPU version took approximately 36 s for HCP and 58.33 min for UKBB. The scaling of computational burden with respect to the number of participants (*N*) was approximately linear for both CPU and GPU versions of FPHI versus ~*N*^2–3^ for GCTA.

## 4. Discussion

We compared the estimates of SNP-heritability (SNP-h^2^) derived using a classical variance component model and empirical coefficients of relatedness (CR) with the SNP-h^2^ estimated from an independent analytic approach using samples of related (Human Connectome Project) and unrelated (UK Biobank) genetic imaging datasets. We showed that heritability estimates obtained using the SOLAR-Eclipse Fast and Powerful Heritability Inference (FPHI) method that was developed to linearize the calculations of the classical heritability model were in good agreement with the estimated provided by the established SNP-h^2^ software - Genome-wide Complex Trait Analysis (GCTA) (Visscher et al., 2006, 2007). We demonstrated an excellent agreement between SNP-*h*^*2*^ values calculated using the FPHI and GCTA and between the results from the HCP and UKBB cohorts, as well as estimates in the UKBB and these reported by the meta-and-mega analysis of heritability studies performed by Enhancing Neuro Imaging Genetics through Meta-Analysis (ENIGMA) consortium. Overall, our findings demonstrated good agreement among genetic contribution to neuroimaging traits regardless of the study/sample design. The small degree of shared genotypic variance in sufficiently large samples such as UKBB can enable standard heritability analyses. We discussed the finding of lower heritability estimates in UKBB versus HCP and attributed it to several well-known factors. Nonetheless, the patterns of additive genetic contribution across the brain were consistent and readily replicable across diverse samples and study designs.

Modern, genetic panels provide the opportunity to directly measure the genetic sharing between any two individuals in a study and calculate the relatedness matrix using empirical, rather than self-reported coefficients of relatedness (CR). Prior work demonstrated that heritability values derived using the empirical CR had better confidence intervals and lower *p*-values as compared to those from analyses using self-reported CR and recommend this approach for genetic analyses in related samples (Kochunov et al., 2019a). GCTA approaches were specifically developed to estimate SNP-h^2^ using from unrelated individuals (Visscher et al., 2006, 2007). However, the SNP-h^2^ estimates by GCTA were shown to be accurate for related samples (Zaitlen et al., 2013). Here, we confirmed that the two methods provided highly consistent (r ~0.9) heritability estimates in datasets of related and unrelated individuals.

We demonstrated significant heritability for a series of neuroanatomical phenotypes that cover structural and diffusion properties of the human brain. We observed an excellent (*r* = 0.7–0.8) agreement in the regional genetic variance across the brain between the HCP and UKBB datasets despite the differences in the study design (twin-siblings versus unrelated), sample size (*N* = ~1000 versus ~37,000) and sample characteristics such as differences in average age (28.8 ± 3.7 versus 63.7 ± 7.5 years for HCP and UKBB respectively) and imaging protocols. The HCP imaging protocol was focused on collecting data at twice (structural) to four (diffusion) times higher spatial resolution than the UKBB images. Despite the differences in protocols, we observed good agreement in the patterns of heritability values among the HCP, UKBB, as well as data published by ENIGMA. This demonstrates that the substantial genetic variance influencing individual differences in brain structure can be readily and consistently measured across diverse samples, study designs, imaging protocols, and software approaches. Importantly, the agreement in the patterns of heritability between UKBB and HCP data provides an opportunity to exploit the greater statistical power of large and inclusive samples such as UKBB for the classical genetic analyses that were previously limited to twins, siblings, and extended pedigree samples.

Despite the excellent agreement in regional patterns, the heritability estimates for the neuroimaging traits in the HCP cohort were approximately twice those (average *h*^*2*^ = 0.72 versus 0.36) observed in the UKBB sample, and for white matter approximately 40% smaller than ENIGMA (average *h*^*2*^ = 0.42 versus 0.67). Likewise, the voxel-wise heritability estimates for the HCP cohort were ~60% higher than those calculated in the UKBB. These absolute differences were independent of the software used to estimate heritability. The SNP-h^2^ values depend on study design, sample characteristics, and the fidelity and ‘closeness’ of the trait to underlying genetic processes. The higher heritability of the neuroimaging traits in the HCP cohort is likely to be the product of three factors: study design, sample differences, and quality of the imaging data. Heritability is the proportion of the variance attributed to the additive genetic variance after correction for covariates. In the HCP sample, we found that sex was the only significant covariate. The HCP sample was designed to reduce the effects of age on the brain measurements by limiting recruitment to an age range that corresponds to a plateau in the brain-aging-versus-development trend (22–35 years) (Kochunov et al., 2011; Van Essen et al., 2013). The focus of UKBB study is on the aging-related disorders, and the age effects were highly significant for all neuroimaging traits in this sample. The lack of aging effects in HCP subjects is the first likely contributor to the higher heritability estimates. The genotype-by-age interaction during aging observed in studies that recruit subjects across the lifespan, can significantly reduce heritability estimates (Batouli et al., 2013; Brouwer et al., 2012, 2020; Glahn et al., 2013).

The HCP study used a twin-sibling recruitment design. Heritability estimates obtained using this study design are typically higher than heritability estimates obtained other study designs such as extended-family-based pedigrees or unrelated samples (Kochunov et al., 2014; Manolio et al., 2009). For instance, heritability measurements of regional white matter traits using self-reported CR HCP were ~20% higher than these estimates reported by ENIGMA studies that combined heritability estimates for cerebral white matter across several world-wide cohorts using meta-analytical and mega-analytical aggregation (Jahanshad et al., 2013; Kochunov et al., 2015). One potential explanation is that the phenotypic variance in complex polygenic traits such as neuroanatomical measurements is also controlled by the heritable epigenetic regulation. This variance is accounted for via study design in the twin-siblings design but less so in extended family and cannot be accounted for in the unrelated sample design (Manolio et al., 2009). One other potential cause of missing heritability is shared early life environment that may shape neuroanatomical traits (Workalemahu et al., 2018). In addition, though there is little variance in age between siblings and none within twin pairs, there is a large variation in the differences in age between pairs of individuals in samples such as the UKBB. Although age is included as a covariate in the model, this correction does not correct for the difference in age between individuals and the impact of this on phenotypic covariance. There is also a possibility that the difference in dataset demographics influences the heritability measures. While there is some variance in ancestry within the UKBB dataset it is a much lower proportion than in the HCP data. The difference in minor allele frequencies between datasets due to these ancestral differences could contribute to the higher heritability within the HCP results, however, this is likely a small contribution as the heritability estimates using self-reported and empirical values showed only minor differences (Kochunov et al., 2015). Lastly, the higher quality of the HCP imaging data likely reduces the measurement error and thus contributes to higher heritability estimates. We note the remarkable agreement in the overall patterns of the regional heritability estimates between the UKBB, HCP, and ENIGMA samples, which argues for the suitability of the UKBB for next-generation genetic analyses focused on understanding imaging genetic networks in complex illnesses.

The SOLAR-Eclipse FPHI is an extension of the standard variance component model that has served the biomedical genetics community for over seven decades. Empirical relatedness is a logical extension of this method, allowing the estimation of additive genetic variation captured by SNP arrays and informative of the genetic architecture of complex traits (Yang et al., 2010). The highly parallel nature of the FPHI algorithm allows for implementation using modern hardware optimized for massively parallel computations of voxel-wise datasets in samples as large as the UKBB. The FPHI code was implemented using linear algebra software libraries that optimize the code for parallel scientific computing in CPU and GPU environments (see supplement section for algorithmic details). This provided a 10^2–4^ -fold acceleration in heritability analyses versus GCTA, which makes the approach especially valuable for studies using data from the UKBB (*N* = 500,000 and growing). The progress of methodological developments in imaging genetics enables the transition from an interrogation of only a few traits to massive voxel-wise analyses in order to study regional variations in genetic influences across the brain.

## 5. Limitations

Empirical CR methods also have a few limitations. The threshold for empirical CR was set at 0 because WAC can produce negative CR values for some unrelated individuals. The negative CR reflect violations of Hardy-Weinberg equilibrium, i.e. ancestral differences in linkage disequilibrium structures, overlapping generations, and deviations from the assumption that genotype frequencies in a population will remain constant from generation to generation (Visscher et al., 2007). GCTA, conversely, retains negative values in the analysis to prevent biases in the iterative likelihood calculations (Visscher et al., 2007). However, we believe that this is a minor limitation, as both methods provided very similar heritability estimates. Empirical CR estimation is sensitive to both the content and quality of genotyping, and this may alter the heritability results. For instance, allowing for more rare variants in the GCTA software led to failure of algorithmic convergence for many traits. Another limitation of this study was the large difference in the number of SNPs between the dataset, as the HCP data were not imputed in accordance with the GCTA guidelines while the only available data from the UKBB had already been imputed. However, we feel that this had little impact on our results and further exemplifies how well the SOLAR FPHI methods agree with the established GTCA methods.

## 6. Conclusion

We show that heritability measurements for complex neuroimaging traits based on empirically measured genetic variance among the largely unrelated participants in the UKBB sample were in agreement with those measured in the twin- and family-based HCP sample. This agreement was observed for both region-based and voxel-wise traits. We likewise observed an excellent agreement between empirical heritability values derived by SOLAR-Eclipse and SNP-*h*^*2*^ values calculated by the GCTA software, suggesting stability of these estimates independent of the analytic methods. Overall, this suggests that large and inclusive samples of unrelated individuals such as data collected by the UKBB can be used to estimate the proportion of phenotypic variance explained by additive genetic factors.

## Supplementary Material

1

## Data Availability

The UK Biobank and Human Connectome Project datasets can be obtained at www.ukbiobank.ac.uk and www.humanconnectome.org/ under a Material Transfer Agreement after presenting a request to the authors describing the intended use of the data. The SOLAR-Eclipse software and tutorial used for the analysis are available for download at www.solar-eclipse-genetics.org. The Genome-wide Complex Trait Analysis software is available for download at cnsgenomics.com/software/gcta/.

## Abbreviations:

CR: coefficients of relatedness
CPU: central processing unit
GPU: graphics processing unit
FPHI: Fast and Powerful Heritability Inference
GCTA: Genome-wide Complex Trait Analysis
UKBB: UK Biobank
HCP: Human Connectome Project
h^2^: heritability
SNP: single nucleotide polymorphism
WAC: weighted allelic correlation
MAF: minor allele frequency
MLE: maximum likelihood estimation
MEGA: Multi-Ethnic Global Array
FA: fractional anisotropy
GRM: genetic relationship matrix
ELRT: expected likelihood ratio test
ENIGMA: Enhancing Neuro Imaging Genetics through Meta-Analysis
GREML: genomic-relatedness-based restricted maximum likelihood
