# Supplementary material for: Comparing empirical kinship derived heritability for imaging genetics traits in the UK biobank and human connectome project
Source: Neuroimage. Author manuscript; Available in PMC 2022 Jan 20. (PMC8771206; doi:10.1016/j.neuroimage.2021.118700)
Supplement: 1 [file NIHMS1770959-supplement-1.docx]

Standard and accelerated heritability measurements

Standard genetic model

Standard computational genetics models to measure heritability and association are based on the variance component analyses and iterative maximization of the likelihood. This approach was created by the father of computational genetics – Ronald Fisher’s original work and has served as the standard analyses for human and animal pedigree analyses (Almasy and Blangero, 1998; Blangero et al., 2001). It uses a linear mixed effect modeling and MLE to compare models with fixed (e.g., the effects of relatedness within the pedigree and genetic variants on the trait) and random variance in the trait. For simplicity, we will assume that effects of fixed covariates such as age, sex, scanner, etc., were removed at the point of preprocessing, and that the phenotype data were normalized such that each trait obeys multivariate normal distribution (Kochunov et al., 2019a). This can be written as Eq. 1, where phenotype Y, such as brain volume per subject, is coded by a vector of length of N (N is the number of subjects) and is assumed to be composed of genetic Yg and environmental Ye parts.

Y= Yg+Ye (1)

Therefore, variance in the trait vector Y can be written as Equation 2:

Variance[Y]= Variance[Yg+Ye] (2)

The variance parameters can be numerically estimated by comparing the observed covariance matrix Ω of Y with the covariance matrices predicted by matrix Φ composed of CR values and identity matrix I that codes for random regimental variance (Almasy and Blangero, 1998) (Eq. 3).

$\Omega=2\sigma_{g}^{2}\Phi+\sigma_{e}^{2}I$ (3)

where σ_e_^2^ is the variance due to individual‑specific environmental effects under the assumption that all environmental effects are uncorrelated among family members e.g. coded by identity matrix. The model in Eq. 1 can be rewritten for association analysis by including the testing of the variance from a SNP_j_ and its beta coefficient (Eq. 4), where the SNPj is a vector (dimension N) of genetic variability for the SNP for each subject, usually coded as 0, 1 or 2 indicating allelic frequency.

Y=SNP_j_· β_j_ + Yg+Ye (4)

This can be rewritten in form of the variance as

Var[Y- SNP_j_· β_j_] = Var[Yg+Ye]= Φ · h^2^ +I·(1- h^2^) (5)

The Eq. 5 is a form of Eq. 3 that was rewritten by using h^2^ as the standard additive genetic heritability or h^2^ = σ^2^_g_/(σ^2^_g_ + σ^2^_e_). The significance of the genetic contribution is tested by comparing the likelihood of the model in which σ_g_^2^ is set to zero with that of a model in which σ_g_^2^ is estimated. Twice the difference between the log_e_ likelihoods of these models yields a test statistic, which is asymptotically distributed as a 1/2:1/2 mixture of a χ^2^ variable with 1 degree-of-freedom and a point mass at zero. The logarithmic function (l) of the likelihood (L) is in Eq 6.

l=ln L = -½ [N⋅ln 2π + ln (Φ · h^2^ +I·( 1- h^2^)) +δ’ ⋅ (Φ · h^2^ +I·( 1- h^2^)))^-1^ ⋅δ] (6)

where δ=Y- SNP_i_ · β_i_. The significance of association is tested by comparing the likelihoods of the model with β_i_ constrained to 0 to the unconstraint model.

For imaging genetic studies, this model (Eq. 6) needs to be evaluated for every trait and every polymorphism. A typical voxel-wise analysis involves ~100,000 traits. Heritability analyses would require 100,000 maximization of the likelihood that can take between 10-50 iterations of typical MLE algorithms. A GWA study of 1,000,000 SNPs would require 10^11^ maximization (number of traits times number of SNPs) of likelihood. Each iteration of the likelihood algorithm requires the inversion of the covariance matrix, Φ · h^2^ +I·(1- h^2^) in Eq. 6. This is a substantial computational effort that goes up by ~*N*^2^ with respect to the number of subjects. These analyses become astronomically complex when using empirical CR matrices in studies such as UK Biobank that has over *N*=45,000 subjects with imaging data. The standard computational genetics model is no longer practical for the massive computational needs required for large-scale imaging-genetics applications. In the next two sections we discuss how such analyses can be made practical using algorithmic and parallel computing capabilities of modern hardware.

SOLAR-Eclipse algorithmic acceleration of standard genetic model

*Minimizing matrix inversion burden*

The first step in making imaging genetic analysis practical is to reduce computational effort associated with the inversion of the covariance matrix for each likelihood calculation in Eq. 6. We proposed the eigen value decomposition (EVD) approach (Blangero et al., 2013) to perform an orthogonal transformation that diagonalizes the covariance (Φ · h^2^ +I·(1- h^2^)) matrix and thus making the matrix inversion trivial. This transform maps the vector Y of non-independent observation to a vector Y^*^ of independent observations. The EVD of the covariance matrix, D_p_ can written as Eq. 7.

V·D_p_·V’ =V· [h^2^·D_g_ + (1- h^2^) ·I] ·V’= V·[I + h^2^ · (D_g_ – I)] ·V’ (7)

where V is the orthogonal matrix of eigenvectors and Dp and Dg are diagonal matrices of phenotypic and genetic eigen values λp and λg. This transformation decorrelates the data for related subjects and reduces the likelihood to the product of univariate normal densities (Blangero et al., 2013). If τ= V’·δ is the vector of residual phenotype values following the transformation to the eigenbasis of the covariance matrix, then the likelihood equation becomes Eq. 8, see (Blangero et al., 2013) for derivations.

l = -½ [ N⋅ln 2π +Σ ln (1+ h^2^·(λg_i_ -1) ) + Στ_i_^2^/(1+ h^2^·(λg_i_ -1))] (8)

Comparing Eq. 6 and Eq. 8, we can see that the likelihood calculations have been simplified to be a sum of univariate likelihoods. This comes with two benefits. The first benefit is that the calculation of the inverse and transformed covariance is now trivial. The second benefit is that the simplified polygenic model can be reduced to simple algebraic solutions for fast approximations calculations that don’t require iterative maximization of the likelihood. We developed two such approximations – to accelerate heritability and association calculations.

*Non-iterative approximations: Two-step Fast and Powerful Heritability Inference (FPHI)*

The solution in Eq. 8 provides a precise estimate of model parameters while greatly reducing ~*N*^2^ burden associated with inversion of the pedigree matrix. However, this solution is still iterative and requires recalculation of likelihood 10-50 times prior to convergence. We developed the FPHI solution for a two-step estimation of heritability. If we don’t consider the variance associated with measured genotypes, then the functional form in Eq. 8 can be simplified as Eq. 9

l (σ^2^_A_, σ^2^_E_) = -½[ N⋅ln 2π + Σ ln ( σ^2^g · λg_i_ + σ^2^e) ) + Στ_i_^2^/(σ^2^g · λg_i_ +σ^2^e))] (9)

where, we again use the definition h^2^ = σ^2^_g_/(σ^2^_g_ + σ^2^_e_), and the covariance matrix becomes σ^2^_g_·Dg + σ^2^_e_I where Dg is a diagonalized matrix of eigenvalues. We define θ as a 2-D vector = (σ^2^_g_, σ^2^_e_). In the standard MLE approach maximization of likelihood is achieved by solving for root value θ_ML_ where l’(θ_ML_) = 0 using the iterative Newton’s method to achieve convergence to θ_ML_

θ_n+1_=θ_n_ – l’(θ_n_) ·l’’(θ_n_)^-1^ (10)

where l’(θ_n_) is the first and l’’(θ_n_) is the second derivatives of the log-likelihood function and n denotes the iteration number. Equation 10 requires inversion of 2x2 l’’(θ_n_) Hessian matrix at every iteration. This becomes a nontrivial computational effort for imaging genetic studies that utilize thousands to hundreds-of-thousands traits.

FPHI method uses a two-step Ordinary Linear Squares (OLS) followed by Weighted Linear Squares (WLS) approximation to solve Eq. 9 non-iteratively (Ganjgahi et al., 2015). If we define a *N*x2 matrix U as [1, λgi]. This amount to solving equation U ·θ=ε^2.^ The OLS solution θ_OLS_= (σ^2^_gOLS_, σ^2^_eOLS_) is given by Eq. 11,

θ_OLS_= max {0 or (U’·U)^-1^·U’·f_OLS_ } (11)

where U is, [1, λgi], an *N*x2 matrix, and ε^2^ is the square of the residual Y*=V’·Y. We set the θ_OLS_ as the OLS ((U’·U)^-1^·U’·f_OLS_), unless it is negative, in which case θ_OLS_ is set to zero to ensure the non-negativity of the solution. This provides a vector and the corresponding h^2^_OLS_ but it is not recommended as a final estimate (Ganjgahi et al., 2015). Instead, the WLS solution given in Eq. 12 is preferred.

θ_WLS_= max { 0 or (U’·(( σ2g_OLS_Dg + σ^2^e_OLS_I)^2^)^-1^·U’·((σ^2^g_OLS_Dg + σ^2^e_OLS_I)^2^)^-1^ · ε^2^_OLS_) } (12)

This WLS estimator is asymptotically normal and unbiased (Ganjgahi et al., 2015). The corresponding heritability estimate is written as Eq. 13

*h^2^*_WLS_ = σ^2^A_WLS_/(σ^2^g_WLS_ + σ^2^e_WLS_) (13)

In the evaluation of both simulated and real data, we showed that *h^2^*_WLS_ provides an excellent approximation for *h^2^*_ML_ as long as the data follows multivariate normal distribution and proposed harmonization strategies to improve agreement (Ganjgahi et al., 2015; Kochunov et al., 2019b).

*CPU vs. GPU implementation of FPHI*

Big data imaging genetic analyses such as voxel-wise heritability calculations in voxel-wise data in HCP and UKBB may benefit from modern computational hardware. The highly parallel and non-iterative nature of the SOLAR-Eclipse FPHI algorithms calls for efficient implementation using modern hardware optimized for massively parallel computations. Contemporary computational clusters are built of nodes equipped with central processing and graphics processing units (CPU/GPU) that offer multiple computational cores (typically 2-64 for CPUs and 500-8000 for GPU). Each core can act as an independent computational unit that can access memory and perform calculations in parallel with other cores. GPUs make parallel computing especially cost effective by offering thousands of computational cores on a single board that is equipped with dedicated high-speed memory. This provides much higher computation power per unit cost. The CPU and GPU versions of FPHI were implemented using linear algebra software libraries that optimize the code for parallel scientific computing in CPU and GPU environments. We used the OpenMP (<https://www.openmp.org>) software library used to implement thread-level parallelization for the CPU version of that software. Within each thread, linear algebra operations were coded using Basic Linear Algebra Subprograms implemented in the Intel Math Kernel Library (<https://software.intel.com/en-us/mkl>).

The GPU algorithms were coded using cuBLAS (<https://developer.nvidia.com/cublas>) linear algebra libraries. The parallelization of scientific calculations for GPU differs from those used in CPU. GPUs utilize many cores for computationally singular operations. A GPU card is optimized for single-instruction, multiple thread (SIMT) processing. This includes such mass parallel tasks as the execution of ray tracing and texture mapping. To give an example of philosophical differences between OpenMP and cuBLAS libraries, let us consider the task of performing a simple math operation on pairs of vectors. The OpenMp approach to the task would be to execute a new parallel thread that contains the code for adding, subtracting or multiplying two vectors. The code within that thread will be executed on a single core and perform operations sequentially on the elements of the vector. A new thread would be issued for each paired operation. The GPU approach differs in that it places the emphasis on operational level parallelization. The GPU cores are not optimized for OpenMP operation that require memory exchange. Instead, the GPU code for addition of two vectors would subdivide the operations to the level of singular operations e.g. adding two elements of a vector and issue this task in a form of a kernel to the GPU board. The addition of multiple vectors will therefore be performed sequentially, rather than in parallel. Typically, scientific algorithms must be redesigned from the ground up to adhere to SIMT architecture of GPU computing, thus making efficient porting of scientific algorithms challenging (Lee et al., 2012). Fortunately, cuBLAS library provides a convenient alternative for porting code written for the OpenMP environment for execution using the GPUs. It also handles parallelization across multiple GPU cards and manages the allocation of global (accessible across GPU), shared (accessible to all threads within a thread block) and register (accessible only to one thread) memory for the developer. The disadvantage of this approach is that it is specific to the devices that support Compute Unified Device Architecture (CUDA). Here, we compared the performance of the CPU and GPU versions of the SOLAR-Eclipse software in HCP and UKBB samples and tested if the computational effort vs. the number of subjects.

| Sample | *N* subjects | N traits | CPU Time per trait (sec) | GPU Time per trait (sec) | CPU total Processing time (sec) | GPU total Processing time (sec) |
| --- | --- | --- | --- | --- | --- | --- |
| HCP | 1,052 | 117,139 | 6.3·10^-4^ | 3.1·10^-4^ | 110 | 36 |
| UKBB | 37,429 | 137,634 | 0.67 | 2.6·10^-2^ | 8.9·10^5^ | 4.1·10^3^ |


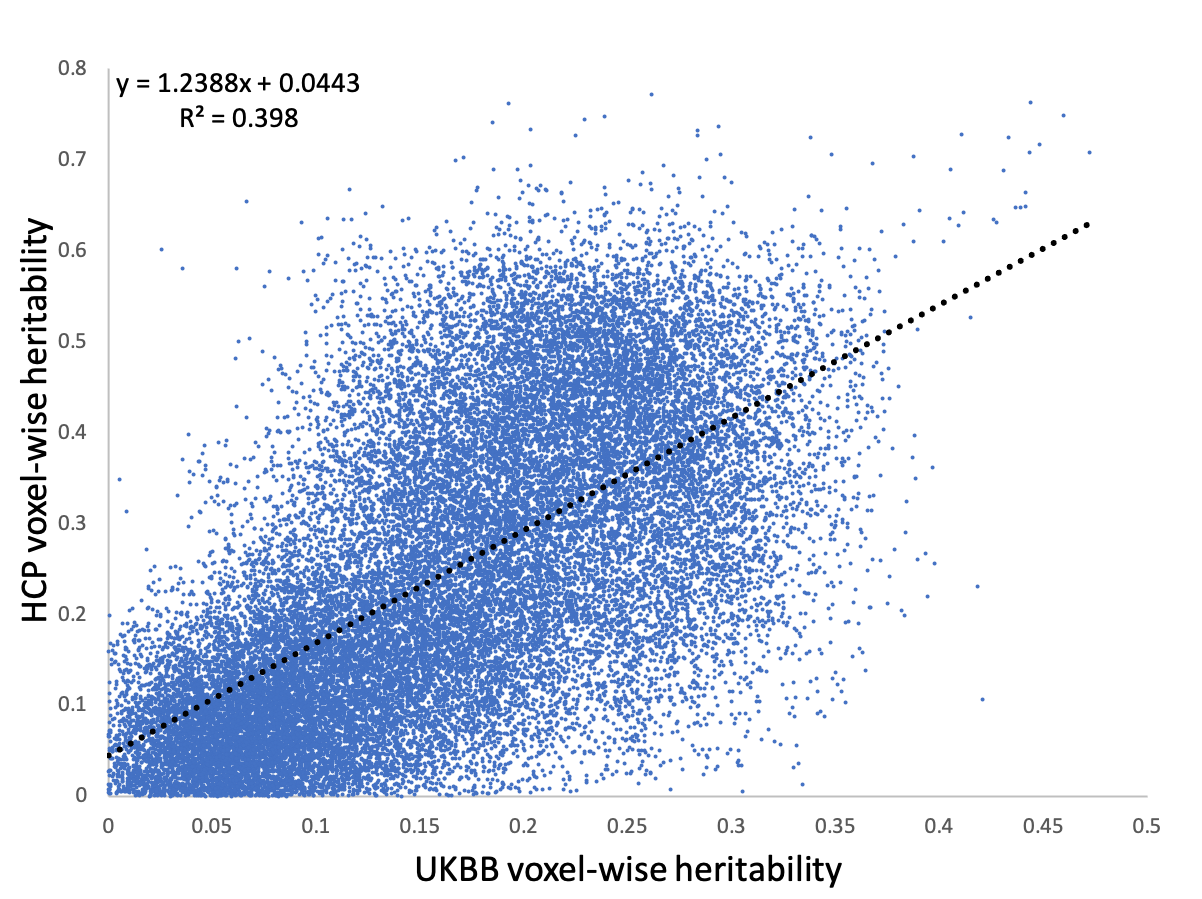


**Figure S1**. Voxel-wise FPHI estimates in the HCP versus UKBB, including a linear fit line, equation, and correlation of determination.

| **Neuroimaging Domains** |  | **Neuroimaging Phenotypes** | **UKBB** | | | | | | | | | | | **HCP** | | | | | | | | | | |
| --- | --- | --- | --- | --- | --- | --- | --- | --- | --- | --- | --- | --- | --- | --- | --- | --- | --- | --- | --- | --- | --- | --- | --- | --- |
|  |  |  | **Age** | | **Sex** | | **Age by Sex** | |  | **FPHI** | | **GCTA** | | **Age** | | **Sex** | | **Age by Sex** | |  | **FPHI** | | **GCTA** | |
|  |  |  | **Estimate** | **P-value** | **Estimate** | **P-value** | **Estimate** | **P-value** | **R-squared** | **h2r** | **P-value** | **Estimate** | **P-value** | **Estimate** | **P-value** | **Estimate** | **P-value** | **Estimate** | **P-value** | **R-squared** | **h2r** | **P-value** | **Estimate** | **P-value** |
| **Cortical Grey Matter Thickness** | 1 | Banks of Superior Temporal Sulcus | -4.01E-03 | <0.001 | -2.40E-02 | <0.001 | 7.65E-08 | <0.001 | 0.05 | 0.27 | 1.01E-64 | 0.21 | 0.00E+00 | -2.92E-03 | 0.03 | 7.66E-02 | 0.18 | -2.17E-03 | 0.27 | 0.02 | 0.63 | 1.60E-31 | 0.53 | 0.00E+00 |
|  | 2 | Caudal Anterior Cingulate Cortex | -4.80E-03 | <0.001 | -1.30E-02 | 0.002 | -4.06E-07 | <0.001 | 0.03 | 0.21 | 1.24E-35 | 0.16 | 0.00E+00 | -2.28E-03 | 0.21 | 1.33E-01 | 0.09 | -5.25E-03 | 0.05 | 0.02 | 0.37 | 1.89E-11 | 0.27 | 2.04E-05 |
|  | 3 | Caudal Middle Frontal Gyrus | -5.35E-03 | <0.001 | -3.34E-02 | <0.001 | 1.34E-07 | <0.001 | 0.11 | 0.35 | 5.56E-94 | 0.28 | 0.00E+00 | -6.51E-03 | <0.001 | 7.61E-02 | 0.15 | -2.57E-03 | 0.15 | 0.07 | 0.8 | 2.85E-55 | 0.79 | 0.00E+00 |
|  | 4 | Cuneus | -5.71E-04 | <0.001 | 1.64E-02 | <0.001 | 8.08E-08 | <0.001 | 0.02 | 0.4 | 2.46E-122 | 0.31 | 0.00E+00 | -5.85E-04 | 0.62 | 3.90E-02 | 0.43 | -1.36E-03 | 0.43 | 0.002 | 0.74 | 3.35E-61 | 0.71 | 0.00E+00 |
|  | 5 | Entorhinal Cortex | -6.98E-03 | <0.001 | -1.25E-02 | <0.001 | -7.56E-08 | <0.001 | 0.04 | 0.2 | 1.90E-34 | 0.15 | 0.00E+00 | 2.23E-04 | 0.92 | -2.00E-02 | 0.84 | 1.06E-03 | 0.76 | 0.001 | 0.36 | 7.60E-17 | 0.29 | 5.00E-14 |
|  | 6 | Frontal Pole | -5.37E-03 | <0.001 | -2.05E-03 | 0.42 | -1.46E-07 | <0.001 | 0.04 | 0.21 | 1.86E-37 | 0.16 | 0.00E+00 | -8.17E-03 | <0.001 | 8.12E-02 | 0.33 | -4.23E-03 | 0.15 | 0.05 | 0.55 | 2.64E-24 | 0.5 | 0.00E+00 |
|  | 7 | Fusiform Gyrus | -4.27E-03 | <0.001 | -1.50E-02 | <0.001 | 3.15E-08 | <0.001 | 0.07 | 0.35 | 7.22E-101 | 0.28 | 0.00E+00 | -1.02E-03 | 0.38 | 5.51E-02 | 0.27 | -1.82E-03 | 0.29 | 0.01 | 0.67 | 3.39E-42 | 0.66 | 0.00E+00 |
|  | 8 | Inferior Parietal Cortex | -5.45E-03 | <0.001 | -2.67E-02 | <0.001 | 6.06E-08 | <0.001 | 0.14 | 0.38 | 7.50E-119 | 0.3 | 0.00E+00 | -3.82E-03 | <0.001 | 1.49E-02 | 0.75 | -7.57E-04 | 0.64 | 0.02 | 0.75 | 6.29E-50 | 0.73 | 0.00E+00 |
|  | 9 | Inferior Temporal Gyrus | -3.20E-03 | <0.001 | 1.48E-02 | <0.001 | 2.22E-08 | 0.001 | 0.04 | 0.33 | 3.76E-96 | 0.25 | 0.00E+00 | -1.47E-03 | 0.24 | 4.45E-02 | 0.41 | -8.42E-04 | 0.65 | 0.01 | 0.68 | 3.98E-36 | 0.61 | 0.00E+00 |
|  | 10 | Insula | -3.68E-03 | <0.001 | 2.23E-02 | <0.001 | -6.34E-08 | <0.001 | 0.03 | 0.29 | 2.23E-69 | 0.23 | 0.00E+00 | -3.20E-03 | 0.01 | 1.56E-01 | 0.01 | -3.12E-03 | 0.1 | 0.11 | 0.51 | 1.89E-20 | 0.5 | 0.00E+00 |
|  | 11 | Isthmus Cingulate Cortex | -4.54E-03 | <0.001 | -9.97E-03 | <0.001 | -1.97E-07 | <0.001 | 0.06 | 0.23 | 4.37E-134 | 0.22 | 0.00E+00 | -6.36E-04 | 0.76 | 2.83E-03 | 0.97 | -2.59E-04 | 0.93 | 0 | 0.27 | 3.43E-07 | 0.12 | 0.00E+00 |
|  | 12 | Lateral Occipital Cortex | -2.41E-03 | <0.001 | 5.16E-03 | <0.001 | 1.27E-07 | <0.001 | 0.05 | 0.42 | 1.66E-139 | 0.32 | 0.00E+00 | -1.82E-04 | 0.87 | 7.74E-02 | 0.11 | -2.33E-03 | 0.16 | 0.01 | 0.69 | 8.22E-42 | 0.78 | 0.00E+00 |
|  | 13 | Lateral Orbitofrontal Cortex | -2.54E-03 | <0.001 | 7.56E-03 | <0.001 | -5.99E-08 | <0.001 | 0.02 | 0.28 | 1.41E-62 | 0.21 | 0.00E+00 | -3.89E-03 | 0 | 1.35E-01 | 0.01 | -4.11E-03 | 0.03 | 0.05 | 0.6 | 3.45E-32 | 0.58 | 4.16E-03 |
|  | 14 | Lingual Gyrus | -1.37E-03 | <0.001 | -5.88E-03 | <0.001 | 1.58E-07 | <0.001 | 0.03 | 0.38 | 1.14E-109 | 0.3 | 0.00E+00 | 4.49E-05 | 0.97 | 6.46E-02 | 0.2 | -1.82E-03 | 0.3 | 0.01 | 0.89 | 3.43E-07 | 0.89 | 0.00E+00 |
|  | 15 | Medial Orbitofrontal Cortex | -3.57E-03 | <0.001 | 2.57E-02 | <0.001 | -1.33E-07 | <0.001 | 0.04 | 0.25 | 9.93E-58 | 0.19 | 0.00E+00 | -2.76E-03 | 0.04 | 9.36E-02 | 0.11 | -2.61E-03 | 0.2 | 0.03 | 0.44 | 2.49E-16 | 0.36 | 0.00E+00 |
|  | 16 | Middle Temporal Gyrus | -4.76E-03 | <0.001 | 2.75E-02 | <0.001 | 1.35E-09 | 0.86 | 0.07 | 0.3 | 3.19E-82 | 0.23 | 0.00E+00 | -3.92E-03 | 0.003 | 5.80E-02 | 0.3 | -1.50E-03 | 0.43 | 0.03 | 0.78 | 5.47E-07 | 0.73 | 0.00E+00 |
|  | 17 | Paracentral Lobule | -7.47E-03 | <0.001 | -1.38E-02 | <0.001 | -5.83E-08 | <0.001 | 0.09 | 0.39 | 2.10E-115 | 0.3 | 0.00E+00 | 2.51E-03 | 0.3 | 1.95E-01 | 0.06 | -7.52E-03 | 0.04 | 0.01 | 0.74 | 5.87E-49 | 0.72 | 4.30E-11 |
|  | 18 | Parahippocampal Gyrus | -5.14E-03 | <0.001 | -4.98E-02 | <0.001 | -2.72E-07 | <0.001 | 0.06 | 0.39 | 8.10E-122 | 0.31 | 0.00E+00 | -2.38E-03 | 0.07 | 5.46E-02 | 0.34 | -2.54E-03 | 0.2 | 0.02 | 0.57 | 3.60E-28 | 0.46 | 0.00E+00 |
|  | 19 | Pars Opercularis of Inferior Frontal Gyrus | -5.05E-03 | <0.001 | 3.78E-03 | 0.02 | 1.06E-07 | <0.001 | 0.1 | 0.3 | 1.34E-72 | 0.27 | 0.00E+00 | -3.88E-03 | 0 | 1.48E-01 | 0.004 | -4.60E-03 | 0.01 | 0.06 | 0.69 | 1.65E-39 | 0.72 | 0.00E+00 |
|  | 20 | Pars Orbitalis of Inferior Frontal Gyrus | -5.32E-03 | <0.001 | -1.76E-02 | <0.001 | -7.57E-08 | <0.001 | 0.07 | 0.24 | 3.01E-50 | 0.19 | 0.00E+00 | -3.73E-03 | 0.01 | 1.34E-01 | 0.03 | -5.11E-03 | 0.02 | 0.03 | 0.55 | 1.28E-24 | 0.52 | 0.00E+00 |
|  | 21 | Pars Triangularis of Inferior Frontal Gyrus | -5.75E-03 | <0.001 | -3.79E-03 | 0.01 | 2.58E-09 | 0.71 | 0.11 | 0.34 | 6.28E-96 | 0.24 | 0.00E+00 | -4.33E-03 | <0.001 | 1.61E-01 | 0.003 | -5.31E-03 | 0 | 0.06 | 0.72 | 3.40E-42 | 0.63 | 0.00E+00 |
|  | 22 | Pericalcarine Cortex | 1.71E-04 | 0.05 | 1.33E-02 | <0.001 | 5.16E-08 | <0.001 | 0.01 | 0.33 | 2.66E-83 | 0.24 | 0.00E+00 | -9.06E-04 | 0.46 | 4.38E-02 | 0.4 | -1.44E-03 | 0.43 | 0 | 0.68 | 1.01E-36 | 0.59 | 0.00E+00 |
|  | 23 | Postcentral Gyrus | -4.51E-03 | <0.001 | -3.77E-02 | <0.001 | 1.50E-07 | <0.001 | 0.09 | 0.39 | 6.26E-116 | 0.31 | 0.00E+00 | -2.03E-04 | 0.85 | 7.87E-02 | 0.09 | -2.75E-03 | 0.09 | 0.01 | 0.92 | 1.43E-07 | 0.8 | 0.00E+00 |
|  | 24 | Posterior Cingulate Cortex | -4.57E-03 | <0.001 | 8.76E-04 | 0.66 | -1.98E-07 | <0.001 | 0.06 | 0.29 | 6.35E-66 | 0.21 | 0.00E+00 | -6.01E-03 | <0.001 | 1.94E-05 | 1 | -1.18E-04 | 0.95 | 0.04 | 0.48 | 3.31E-18 | 0.39 | 0.00E+00 |
|  | 25 | Precentral Gyrus | -7.41E-03 | <0.001 | -2.54E-02 | <0.001 | 7.53E-08 | <0.001 | 0.12 | 0.37 | 5.42E-103 | 0.28 | 0.00E+00 | -3.57E-03 | 0.003 | 4.86E-02 | 0.34 | -1.48E-03 | 0.4 | 0.03 | 0.84 | 3.51E-62 | 0.83 | 0.00E+00 |
|  | 26 | Precuneus | -5.26E-03 | <0.001 | -2.61E-02 | <0.001 | 1.26E-07 | <0.001 | 0.12 | 0.39 | 1.90E-116 | 0.31 | 0.00E+00 | -3.24E-03 | 0.01 | 3.66E-02 | 0.46 | -1.31E-03 | 0.44 | 0.02 | 0.71 | 1.36E-47 | 0.71 | 5.05E-12 |
|  | 27 | Rostral Anterior Cingulate Cortex | -4.17E-03 | <0.001 | 3.33E-02 | <0.001 | -3.80E-07 | <0.001 | 0.05 | 0.24 | 1.57E-50 | 0.18 | 0.00E+00 | -2.38E-03 | 0.18 | 1.59E-01 | 0.04 | -4.66E-03 | 0.08 | 0.02 | 0.38 | 1.84E-12 | 0.36 | 0.00E+00 |
|  | 28 | Rostral Middle Frontal Gyrus | -5.71E-03 | <0.001 | 4.82E-03 | <0.001 | 2.45E-09 | 0.69 | 0.14 | 0.36 | 1.06E-106 | 0.29 | 0.00E+00 | -4.45E-03 | <0.001 | 1.32E-01 | 0.01 | -4.42E-03 | 0.01 | 0.05 | 0.77 | 8.47E-53 | 0.76 | 0.00E+00 |
|  | 29 | Superior Frontal Gyrus | -7.73E-03 | <0.001 | -1.83E-02 | <0.001 | -3.78E-08 | <0.001 | 0.17 | 0.37 | 6.34E-107 | 0.31 | 0.00E+00 | -6.43E-03 | <0.001 | 8.48E-02 | 0.14 | -3.69E-03 | 0.06 | 0.06 | 0.83 | 1.76E-60 | 0.85 | 1.26E-10 |
|  | 30 | Superior Parietal Cortex | -4.64E-03 | <0.001 | -3.35E-02 | <0.001 | 1.34E-07 | <0.001 | 0.11 | 0.38 | 2.18E-109 | 0.31 | 0.00E+00 | -1.06E-03 | 0.33 | 6.76E-02 | 0.14 | -2.71E-03 | 0.09 | 0.01 | 0.8 | 2.23E-59 | 0.79 | 0.00E+00 |
|  | 31 | Superior Temporal Gyrus | -7.31E-03 | <0.001 | -3.90E-03 | 0.04 | 1.21E-07 | <0.001 | 0.14 | 0.37 | 6.94E-111 | 0.3 | 0.00E+00 | -2.50E-03 | 0.06 | 1.12E-01 | 0.05 | -2.65E-03 | 0.17 | 0.04 | 0.72 | 1.88E-49 | 0.69 | 0.00E+00 |
|  | 32 | Supramarginal Gyrus | -5.90E-03 | <0.001 | -2.77E-02 | <0.001 | 4.28E-08 | <0.001 | 0.13 | 0.38 | 7.08E-114 | 0.3 | 0.00E+00 | -3.41E-03 | 0.004 | 7.42E-02 | 0.14 | -2.95E-03 | 0.09 | 0.03 | 0.78 | 1.13E-56 | 0.75 | 0.00E+00 |
|  | 33 | Transverse Temporal Gyrus | 2.13E-05 | 0.89 | -1.79E-02 | <0.001 | 5.45E-08 | <0.001 | 0.001 | 0.33 | 1.46E-88 | 0.26 | 0.00E+00 | -1.43E-03 | 0.39 | 4.84E-02 | 0.5 | -1.84E-03 | 0.45 | 0 | 0.69 | 1.76E-60 | 0.58 | 2.81E-13 |
| **Subcortical Volume** | 34 | Accumbens | -4.97E+00 | <0.001 | -4.49E+00 | <0.001 | 3.23E-04 | <0.001 | 0.29 | 0.29 | 2.82E-70 | 0.23 | 0.00E+00 | -3.55E+00 | <0.001 | 2.36E+01 | 0.54 | 1.78E+00 | 0.18 | 0.21 | 0.74 | 4.51E-45 | 0.78 | 0.00E+00 |
|  | 35 | Amygdala | 9.19E-01 | <0.001 | 6.01E+01 | <0.001 | 5.89E-04 | <0.001 | 0.16 | 0.29 | 2.55E-42 | 0.25 | 0.00E+00 | -2.33E+00 | 0.21 | 2.32E+02 | 0.003 | -2.89E-02 | 0.99 | 0.34 | 0.85 | 7.99E-65 | 0.89 | 0.00E+00 |
|  | 36 | Caudate | 5.37E-01 | 0.03 | -2.06E+01 | <0.001 | 2.25E-03 | <0.001 | 0.35 | 0.52 | 3.80E-207 | 0.44 | 0.00E+00 | -1.99E+01 | <0.001 | -1.92E+02 | 0.37 | 1.80E+01 | 0.01 | 0.14 | 0.89 | 6.01E-77 | 0.88 | 0.00E+00 |
|  | 37 | Hippocampus | -1.18E+01 | <0.001 | 4.00E+00 | 0.41 | 1.67E-03 | <0.001 | 0.26 | 0.3 | 8.68E-72 | 0.23 | 0.00E+00 | -2.10E+00 | 0.64 | 2.82E+02 | 0.14 | 5.86E+00 | 0.37 | 0.25 | 0.78 | 0.5 | 0.87 | 0.00E+00 |
|  | 38 | Pallidum | -1.80E+00 | <0.001 | 2.10E+01 | <0.001 | 9.82E-04 | <0.001 | 0.28 | 0.34 | 1.52E-95 | 0.28 | 0.00E+00 | -8.69E+00 | <0.001 | 1.38E+02 | 0.12 | 1.22E-01 | 0.97 | 0.17 | 0.69 | 6.73E-37 | 0.72 | 0.00E+00 |
|  | 39 | Putamen | -1.53E+01 | <0.001 | 1.37E+02 | <0.001 | 2.79E-03 | <0.001 | 0.47 | 0.46 | 3.35E-170 | 0.39 | 0.00E+00 | -3.04E+01 | <0.001 | 3.47E+02 | 0.21 | 7.70E+00 | 0.42 | 0.24 | 0.88 | 3.44E-69 | 0.88 | 0.00E+00 |
|  | 40 | Thalamus | -1.96E+01 | <0.001 | 6.76E+00 | 0.27 | 4.61E-03 | <0.001 | 0.6 | 0.48 | 3.26E-175 | 0.38 | 0.00E+00 | -1.97E+01 | 0.01 | 8.87E+01 | 0.8 | 2.45E+01 | 0.04 | 0.24 | 0.79 | 1.59E-54 | 0.81 | 0.00E+00 |
| **White Matter FA Tracts** | 41 | Anterior Corona Radiata (ACR) | -1.69E-03 | <0.001 | -4.02E-03 | <0.001 | 5.12E-09 | 0.001 | 0.18 | 0.47 | 3.59E-181 | 0.41 | 0.00E+00 | -5.01E-04 | 0.09 | 6.15E-03 | 0.62 | -2.32E-04 | 0.59 | 0.01 | 0.82 | 1.37E-54 | 0.83 | 0.00E+00 |
|  | 42 | Anterior Limb of Internal Capsule (ALIC) | -6.30E-04 | <0.001 | 3.16E-03 | <0.001 | 2.71E-08 | <0.001 | 0.07 | 0.45 | 4.62E-160 | 0.38 | 0.00E+00 | 3.75E-05 | 0.88 | -8.04E-03 | 0.46 | -1.08E-04 | 0.77 | 0.06 | 0.8 | 1.34E-50 | 0.8 | 0.00E+00 |
|  | 43 | Body of Corpus Callosum (BCC) | -1.04E-03 | <0.001 | -5.07E-03 | <0.001 | 2.65E-08 | <0.001 | 0.09 | 0.44 | 8.15E-151 | 0.36 | 0.00E+00 | -5.97E-04 | 0.05 | -1.69E-02 | 0.19 | -4.87E-05 | 0.91 | 0.1 | 0.8 | 3.79E-53 | 0.9 | 0.00E+00 |
|  | 44 | Cingulum (CGC) | -9.21E-04 | <0.001 | 3.41E-03 | <0.001 | 4.68E-08 | <0.001 | 0.1 | 0.48 | 3.29E-177 | 0.41 | 0.00E+00 | -1.67E-04 | 0.59 | -5.47E-03 | 0.67 | -2.07E-04 | 0.65 | 0.04 | 0.78 | 2.23E-47 | 0.81 | 0.00E+00 |
|  | 45 | Cortico-Spinal Tract (CST) | -6.38E-04 | <0.001 | 1.04E-02 | <0.001 | 4.94E-08 | <0.001 | 0.11 | 0.29 | 5.51E-67 | 0.23 | 0.00E+00 | -2.72E-04 | 0.52 | -2.17E-02 | 0.23 | 2.27E-04 | 0.72 | 0.04 | 0.65 | 2.55E-31 | 0.61 | 0.00E+00 |
|  | 46 | External Capsule (EC) | -8.96E-04 | <0.001 | -5.56E-03 | <0.001 | 5.35E-08 | <0.001 | 0.16 | 0.42 | 4.44E-136 | 0.36 | 0.00E+00 | -1.50E-05 | 0.94 | -1.34E-02 | 0.14 | 1.16E-04 | 0.71 | 0.07 | 0.78 | 3.15E-48 | 0.87 | 0.00E+00 |
|  | 47 | Fornix (FX) | -5.87E-03 | <0.001 | -2.43E-02 | <0.001 | -3.75E-08 | <0.001 | 0.28 | 0.32 | 2.60E-83 | 0.26 | 0.00E+00 | 4.28E-04 | 0.24 | -1.52E-02 | 0.32 | -1.37E-04 | 0.79 | 0.09 | 0.53 | 1.38E-20 | 0.53 | 0.00E+00 |
|  | 48 | Genu of Corpus Callosum (GCC) | -1.44E-03 | <0.001 | -5.51E-03 | <0.001 | 4.42E-08 | <0.001 | 0.13 | 0.41 | 2.58E-135 | 0.34 | 0.00E+00 | -1.19E-03 | 4.48E-04 | -1.53E-02 | 0.28 | 2.69E-04 | 0.59 | 0.03 | 0.89 | 1.85E-72 | 0.91 | 0.00E+00 |
|  | 49 | Posterior Corona Radiata (PCR) | -5.17E-04 | <0.001 | -1.71E-03 | <0.001 | 4.55E-09 | 0.001 | 0.03 | 0.42 | 1.13E-136 | 0.34 | 0.00E+00 | -2.88E-04 | 0.27 | -8.95E-03 | 0.42 | 2.00E-05 | 0.96 | 0.03 | 0.82 | 2.85E-58 | 0.85 | 0.00E+00 |
|  | 50 | Posterior Limb of Internal Capsule (PLIC) | -1.15E-04 | <0.001 | 3.43E-04 | 0.25 | 3.21E-08 | <0.001 | 0.03 | 0.47 | 3.00E-163 | 0.38 | 0.00E+00 | -2.76E-04 | 0.29 | -1.38E-02 | 0.21 | -1.76E-04 | 0.64 | 0.15 | 0.85 | 1.66E-60 | 0.87 | 0.00E+00 |
|  | 51 | Retrolenticular Limb of the Internal Capsule (RLIC) | -3.29E-04 | <0.001 | -1.57E-03 | <0.001 | 3.40E-08 | <0.001 | 0.03 | 0.43 | 2.36E-141 | 0.34 | 0.00E+00 | -2.31E-04 | 0.44 | -2.31E-02 | 0.07 | 1.87E-04 | 0.67 | 0.11 | 0.82 | 3.59E-56 | 0.84 | 0.00E+00 |
|  | 52 | Internal Capsule (IC) | -3.58E-04 | <0.001 | 6.47E-04 | 0.01 | 3.10E-08 | <0.001 | 0.06 | 0.45 | 7.87E-142 | 0.37 | 0.00E+00 | -1.63E-04 | 0.49 | -1.49E-02 | 0.14 | -4.10E-05 | 0.91 | 0.13 | 0.87 | 8.01E-67 | 0.89 | 0.00E+00 |
|  | 53 | Sagittal Striatum (SS) | -8.41E-04 | <0.001 | -3.96E-03 | <0.001 | -3.63E-09 | 0.03 | 0.05 | 0.41 | 3.78E-130 | 0.35 | 0.00E+00 | -1.43E-04 | 0.63 | -1.77E-02 | 0.15 | 8.28E-05 | 0.85 | 0.08 | 0.83 | 7.75E-55 | 0.85 | 0.00E+00 |
|  | 54 | Splenium of Corpus Callosum (SCC) | -2.93E-04 | <0.001 | -2.35E-04 | 0.41 | 1.06E-09 | 0.4 | 0.01 | 0.37 | 1.25E-112 | 0.3 | 0.00E+00 | -3.65E-04 | 0.16 | -1.64E-02 | 0.14 | 5.66E-05 | 0.88 | 0.1 | 0.87 | 3.79E-67 | 0.91 | 0.00E+00 |
|  | 55 | Superior Corona Radiata (SCR) | -6.82E-04 | <0.001 | -5.72E-03 | <0.001 | 3.41E-08 | <0.001 | 0.08 | 0.45 | 3.56E-153 | 0.37 | 0.00E+00 | -3.42E-04 | 0.16 | 3.86E-03 | 0.71 | -3.99E-04 | 0.27 | 0.03 | 0.8 | 3.79E-67 | 0.77 | 0.00E+00 |
|  | 56 | Superior Longetudinal Fasciculus (SLF) | -6.51E-04 | <0.001 | -1.71E-03 | <0.001 | 1.64E-08 | <0.001 | 0.05 | 0.48 | 3.68E-176 | 0.4 | 0.00E+00 | -3.87E-04 | 0.12 | -6.57E-03 | 0.53 | 8.12E-05 | 0.82 | 0.01 | 0.84 | 2.83E-59 | 0.89 | 0.00E+00 |
|  | 57 | Posterior Thalamic Radiation (PTR) | -1.54E-03 | <0.001 | -4.89E-03 | <0.001 | -2.31E-08 | <0.001 | 0.13 | 0.39 | 7.09E-119 | 0.32 | 0.00E+00 | -4.66E-04 | 0.07 | -1.69E-02 | 0.12 | 2.43E-04 | 0.52 | 0.05 | 0.85 | 3.15E-48 | 0.88 | 0.00E+00 |
|  | 58 | Superior Fronto-Occipital Fasciculus (SFO) | -1.90E-03 | <0.001 | -2.92E-03 | <0.001 | 2.54E-08 | <0.001 | 0.13 | 0.43 | 1.64E-146 | 0.35 | 0.00E+00 | -1.32E-04 | 0.67 | -6.59E-03 | 0.62 | 2.83E-05 | 0.95 | 0.01 | 0.76 | 3.15E-48 | 0.75 | 0.00E+00 |
|  | 59 | Uncinate Fasciculus (UNC) | -5.21E-04 | <0.001 | 8.86E-04 | 0.04 | 4.22E-08 | <0.001 | 0.04 | 0.45 | 2.29E-185 | 0.39 | 0.00E+00 | -7.50E-05 | 0.88 | -2.40E-02 | 0.24 | 1.79E-04 | 0.8 | 0.05 | 0.74 | 1.80E-43 | 0.85 | 0.00E+00 |
|  | 60 | Tapetum (TAP) | -2.03E-03 | <0.001 | -5.87E-03 | <0.001 | -8.04E-08 | <0.001 | 0.08 | 0.42 | 1.77E-142 | 0.35 | 0.00E+00 | -5.81E-04 | 0.24 | -3.27E-02 | 0.12 | 1.07E-03 | 0.14 | 0.003 | 0.79 | 2.90E-37 | 0.69 | 0.00E+00 |

**Table S1.** Heritability and effects of age, sex and age by sex interaction on the UKBB and HCP datasets.

|  | **FPHI** | | **GCTA** | | **ENIGMA** | | | |
| --- | --- | --- | --- | --- | --- | --- | --- | --- |
|  | **h2** | **P-value** | **Estimate** | **P-value** | **MetaAnalysis (SE)** | **P-value** | **Mega Analysis** | **P-value** |
| AverageFA | 0.43 |  |  |  | 0.71 | <1E-10 | 0.67 | <1E-10 |
| BCC | 0.44 | 8.15E-151 | 0.36 | <1E-10 | 0.65 | <1E-10 | 0.69 | <1E-10 |
| GCC | 0.41 | 2.58E-135 | 0.34 | <1E-10 | 0.75 | <1E-10 | 0.66 | <1E-10 |
| SCC | 0.37 | 1.25E-112 | 0.30 | <1E-10 | 0.66 | <1E-10 | 0.62 | <1E-10 |
| FX | 0.32 | 2.6E-83 | 0.26 | <1E-10 | 0.49 | <1E-10 | 0.56 | <1E-10 |
| CGC | 0.48 | 3.29E-177 | 0.41 | <1E-10 | 0.66 | <1E-10 | 0.63 | <1E-10 |
| CR | 0.45 |  | 0.38 |  | 0.73 | <1E-10 | 0.75 | <1E-10 |
| EC | 0.42 | 4.44E-136 | 0.36 | <1E-10 | 0.77 | <1E-10 | 0.75 | <1E-10 |
| IC | 0.45 |  | 0.37 |  | 0.71 | <1E-10 | 0.71 | <1E-10 |
| TAP | 0.45 | 2.29E-185 | 0.35 | <1E-10 | 0.71 | <1E-10 | 0.73 | <1E-10 |
| PTR | 0.39 | 7.09E-119 | 0.32 | <1E-10 | 0.76 | <1E-10 | 0.69 | <1E-10 |
| SFO | 0.43 | 1.64E-146 | 0.35 | <1E-10 | 0.62 | <1E-10 | 0.64 | <1E-10 |
| SLF | 0.48 | 3.68E-176 | 0.40 | <1E-10 | 0.82 | 0 | 0.77 | <1E-10 |
| SS | 0.41 | 3.78E-130 | 0.35 | <1E-10 | 0.66 | <1E-10 | 0.65 | <1E-10 |
| CST | 0.29 | 5.51E-67 | 0.23 | <1E-10 | 0.44 | <1E-10 | 0.42 | <1E-10 |
| UNC | 0.45 | 2.29E-185 | 0.39 | <1E-10 | 0.78 | <1E-10 | 0.71 | <1E-10 |

**Table S2.** FPHI and GCTA estimates vs ENIGMA in UKBB.

|  | **UKBB (*N*=37,432)** | **HCP (*N*=1,206)** |
| --- | --- | --- |
|  | mean (SD), range | mean (SD), range |
| Age | 63.7 (7.5), 45~82 | 28.8 (3.7), 22~37 |
|  | *N* (percentage) | *N* (percentage) |
| Sex: Male | 17531 (46.8%) | 550 (45.6%) |
| Sex: Female | 19901 (53.2%) | 656 (54.4%) |
| N: number of subjects, SD: Standard Deviation | | |

**Table S3.** Demographics in the UKBB and HCP

References

Almasy, L., & Blangero, J. (1998). Multipoint quantitative-trait linkage analysis in general pedigrees. *Am J Hum Genet*, *62*(5), 1198-1211. <https://doi.org/10.1086/301844>

Blangero, J., Diego, V. P., Dyer, T. D., Almeida, M., Peralta, J., Kent, J. W., Jr., Williams, J. T., Almasy, L., & Goring, H. H. (2013). A kernel of truth: statistical advances in polygenic variance component models for complex human pedigrees. *Adv Genet*, *81*, 1-31. <https://doi.org/10.1016/B978-0-12-407677-8.00001-4>

Blangero, J., Williams, J. T., & Almasy, L. (2001). Variance component methods for detecting complex trait loci. *Adv Genet*, *42*, 151-181. <https://doi.org/10.1016/s0065-2660(01)42021-9>

Ganjgahi, H., Wincker, A., DC., G., Blangero, J., P., K., & Nichols, T. (2015). Fast and Powerful Heritability Inference for Family-Based Neuroimaging Studies. *Neuroimage*, *115*, 256-268. <https://doi.org/10.1016/j.neuroimage.2015.03.005>

Kochunov, P., Donohue, B., Mitchell, B. D., Ganjgahi, H., Adhikari, B., Ryan, M., Medland, S. E., Jahanshad, N., Thompson, P. M., Blangero, J., Fieremans, E., Novikov, D. S., Marcus, D., Van Essen, D. C., Glahn, D. C., Elliot Hong, L., & Nichols, T. E. (2019a). Genomic kinship construction to enhance genetic analyses in the human connectome project data. *Human Brain Mapping*, *40*(5), 1677-1688. <https://doi.org/10.1002/hbm.24479>

Kochunov, P., Patel, B., Ganjgahi, H., Donohue, B., Ryan, M., Hong, E. L., Chen, X., Adhikari, B., Jahanshad, N., Thompson, P. M., Vanâ€™t Ent, D., den Braber, A., de Geus, E. J. C., Brouwer, R. M., Boomsma, D. I., Hulshoff Pol, H. E., de Zubicaray, G. I., McMahon, K. L., Martin, N. G., . . . Nichols, T. E. (2019b). Homogenizing Estimates of Heritability Among SOLAR-Eclipse, OpenMx, APACE, and FPHI Software Packages in Neuroimaging Data [Original Research]. *Frontiers in Neuroinformatics*, *13*(16). <https://doi.org/10.3389/fninf.2019.00016>

Lee, D., Dinov, I., Dong, B., Gutman, B., Yanovsky, I., & Toga, A. W. (2012). CUDA optimization strategies for compute- and memory-bound neuroimaging algorithms. *Comput Methods Programs Biomed*, *106*(3), 175-187. <https://doi.org/10.1016/j.cmpb.2010.10.013>
